# Upconversion of Cellulosic Waste Into a Potential “Drop in Fuel” via Novel Catalyst Generated Using *Desulfovibrio desulfuricans* and a Consortium of Acidophilic Sulfidogens

**DOI:** 10.3389/fmicb.2019.00970

**Published:** 2019-05-10

**Authors:** Iryna P. Mikheenko, Jaime Gomez-Bolivar, Mohamed L. Merroun, Lynne E. Macaskie, Surbhi Sharma, Marc Walker, Rachel A. Hand, Barry M. Grail, David Barrie Johnson, Rafael L. Orozco

**Affiliations:** ^1^School of Biosciences, University of Birmingham, Birmingham, United Kingdom; ^2^Department of Microbiology, Faculty of Sciences, University of Granada, Granada, Spain; ^3^Department of Physics, University of Warwick, Coventry, United Kingdom; ^4^Department of Chemistry, University of Warwick, Coventry, United Kingdom; ^5^School of Natural Sciences, Bangor University, Gwynedd, United Kingdom

**Keywords:** 5-hydroxymethylfurfural upgrade, 5-HMF upgrade, PdRu catalyst, *Desulfovibrio desulfuricans*, waste sulfidogenic bacteria

## Abstract

Biogas-energy is marginally profitable against the “parasitic” energy demands of processing biomass. Biogas involves microbial fermentation of feedstock hydrolyzate generated enzymatically or thermochemically. The latter also produces 5-hydroxymethyl furfural (5-HMF) which can be catalytically upgraded to 2, 5-dimethyl furan (DMF), a “drop in fuel.” An integrated process is proposed with side-stream upgrading into DMF to mitigate the “parasitic” energy demand. 5-HMF was upgraded using bacterially-supported Pd/Ru catalysts. Purpose-growth of bacteria adds additional process costs; Pd/Ru catalysts biofabricated using the sulfate-reducing bacterium (SRB) *Desulfovibrio desulfuricans* were compared to those generated from a waste consortium of acidophilic sulfidogens (CAS). Methyl tetrahydrofuran (MTHF) was used as the extraction-reaction solvent to compare the use of bio-metallic Pd/Ru catalysts to upgrade 5-HMF to DMF from starch and cellulose hydrolyzates. MTHF extracted up to 65% of the 5-HMF, delivering solutions, respectively, containing 8.8 and 2.2 g 5-HMF/L MTHF. Commercial 5% (wt/wt) Ru-carbon catalyst upgraded 5-HMF from pure solution but it was ineffective against the hydrolyzates. Both types of bacterial catalyst (5wt%Pd/3-5wt% Ru) achieved this, bio-Pd/Ru on the CAS delivering the highest conversion yields. The yield of 5-HMF from starch-cellulose thermal treatment to 2,5 DMF was 224 and 127 g DMF/kg extracted 5-HMF, respectively, for CAS and *D. desulfuricans* catalysts, which would provide additional energy of 2.1 and 1.2 kWh/kg extracted 5-HMF. The CAS comprised a mixed population with three patterns of metallic nanoparticle (NP) deposition. Types I and II showed cell surface-localization of the Pd/Ru while type III localized NPs throughout the cell surface and cytoplasm. No metallic patterning in the NPs was shown via elemental mapping using energy dispersive X-ray microanalysis but co-localization with sulfur was observed. Analysis of the cell surfaces of the bulk populations by X-ray photoelectron spectroscopy confirmed the higher S content of the CAS bacteria as compared to *D. desulfuricans* and also the presence of Pd-S as well as Ru-S compounds and hence a mixed deposit of PdS, Pd(0), and Ru in the form of various +3, +4, and +6 oxidation states. The results are discussed in the context of recently-reported controlled palladium sulfide ensembles for an improved hydrogenation catalyst.

## Introduction

The climatic impact of atmospheric CO_2,_ a legacy of the use of fossil fuels, is now accepted and stricter worldwide environmental legislation has promoted global interest in developing carbon-neutral fuels from biomass (Chu and Majumdar, 2012; Al-Amin et al., 2015; Cadez and Czerny, 2016), consistent with increasing value creation from natural resources (e.g., renewable biomass) within the concept of a circular economy (Govindan and Hasanagic, 2018).

Biomass sources include wood, plants, agricultural and energy crops, aquatic plants and food processing wastes, e.g., stems and husks. Although biomass-derived fuels offer a renewable and sustainable potential alternative to fossil fuels biomass conversion technologies are usually needed. These are generally grouped into two categories: biochemical and thermochemical. The former depends on the relatively slow action of microorganisms and/or enzymatic catalysts at moderate temperatures (e.g., up to ∼60°C) which usually follow a mechanical, thermal or chemical pre-treatment of the native biomass (Modenbach and Nokes, 2013; Haldar et al., 2016). The latter require high temperatures and pressures (e.g., 200–375°C and 40–220 bar, respectively) with or without the presence of metallic/inorganic-catalysts to obtain products from different biomass sources (Gollakota et al., 2018) in a matter of hours. Less aggressive methods such as the Hydro Thermal Hydrolysis (HTH) processes (e.g., Dogaris et al., 2009; Vardon et al., 2012) provide a route for wet biomass (e.g., algae Kunwar et al., 2017) conversion, forestalling the energy demand of drying. The process uses water as the reaction solvent, this being compatible with downstream fermentation of the product.

Starch and cellulose are the predominant polymeric materials in biomass. Depending on the reaction conditions their HTH generates hydrolyzates containing mainly sugars (e.g., glucose, fructose) for onward fermentation into gaseous fuels, significant amounts of 5-hydroxymethylfurfural (5-HMF; a fermentation inhibitor), and smaller amounts of other sub-products resulting from further degradation of 5-HMF during the reaction (Palmqvist and Hahn-Hägerdal, 2000). The sugars are readily fermented (e.g., to make biohydrogen) following toxic 5-HMF removal (Orozco, 2012; Redwood et al., 2012), however, at the same time the latter co-product can provide a potential resource in parallel to the primary fermentation process.

5-HMF is a versatile platform chemical that offers potential pathways to the synthesis of valuable products including polymers, fine chemicals and biofuels (Yang et al., 2017). Hence it is important to explore and evaluate these routes to mitigate the shortfall of the HTH process economics mainly attributed to the high-heat requirement (Gollakota et al., 2018) for the thermochemical biomass processing, as well as the high power demand of biomass comminution upstream. For example the energy consumption to mill *Miscanthus* (moisture content of 15%) to 4 mm was determined at 184 kJ/kg of dry matter (Miao et al., 2011). Gollakota et al. (2018) noted that an efficient algal feedstock-HTH process (@ 280°C, 15 min) consumed ∼15% of the energy contained in the feedstock thereby yielding a potential energy efficiency of ∼85% (Gollakota et al., 2018). By using algal biomass comminution is not required. Other studies calculated an energy efficiency of 63.9% for thermal hydrolysis (300°C) in a cornstalk-HTH (Shi et al., 2013).

Among the possibilities for 5-HMF conversion into valuable products 2,5 dimethylfuran (DMF) is a biofuel of particular importance due to its high energy density (30 MJ/L) (similar to gasoline: 31.9 MJ/L), its high octane number, low oxygen content (O/C 0.17), its immiscibility with water and its affinity to blend with fossil-derived fuels and ethanol (Roman-Leshkov et al., 2007; Thananatthanachon and Rauchfuss, 2010; Zhang et al., 2017) as well as its proven use in a direct-injection spark-ignition engine (Dang et al., 2016). DMF is not water soluble, has a boiling point of 92–96°C and its evaporation requires approximately one-third less energy than the evaporation of ethanol (Da Silva and Aznar, 2014) which is widely used as a biofuel despite the energy demand of distillation.

The catalytic upgrading of 5-HMF to DMF, proposed as a route to making liquid fuel from carbohydrates (Roman-Leshkov et al., 2007) proceeds in the absence of water as the latter negatively impacts in the hydrogenation reactions, decreasing yields and selectivity (Liu et al., 2015). HTH is performed in an aqueous system, hence an ideal method would both separate the 5-HMF from the fermentable aqueous phase (detoxifying it) and maximize its catalytic upgrading to DMF. The separation of 5-HMF from the hydrolysis products in the aqueous phase is a challenge that must be overcome in order to detoxify the fermentation stream and valorize the 5-HMF component into local power, with the extraction and catalytic upgrading steps in a common solvent. The selection and evaluation of solvent for this dual role was the first aim of the study, considering two main factors: The 5-HMF-solvent partition coefficient (P_HMF_ [wt%_org_/wt%_aq_]) and the solvent compatibility with the catalytic upgrading reactions. Partition coefficient (P_HMF_) quantifies the equilibrium distribution of a solute between 2 immiscible phases and is a measure for solvent extraction efficiency. The higher the P_HMF_ value the higher the extraction efficiency.

Tetrahydrofuran (THF) is an efficient solvent for the catalytic transformation of 5-HMF to DMF in the presence of ruthenium catalysts, delivering DMF yields up to 95% (Hu et al., 2014). However, its miscibility with water limits its application here. Methyl tetrahydrofuran (MTHF) is a solvent produced from renewable resources (Aycock, 2007) with similar properties to THF [relatively high partition coefficient (P_HMF_ of 2.1)] and low water solubility (4 g/100 mL)]. MTHF has replaced THF in several organometallic-catalyzed reactions (Aycock, 2007; Blumenthal et al., 2016). Moreover, the presence of sugars (glucose and fructose) in the hydrolyzate can enhance the extraction capacity of the MTHF and induce phase separation. For example, with the addition of 10, 30, or 50 wt% of fructose the partition coefficient increased by >40–50% for MTHF (Blumenthal et al., 2016). MTHF delivers clean organic-water phase separations and, unlike THF, it can be used to dry the product for a subsequent reaction or isolation step (Aycock, 2007).

The second focus of this study is the catalytic upgrading of 5-HMF to DMF and the scope for using novel biogenic metal catalysts for this reaction. Other work reports that ruthenium catalyst can achieve this conversion (Hu et al., 2014; Lei et al., 2014; Nagpure et al., 2015). Hu et al. (2014) reported DMF yields of 95% while Nagpure et al. (2015) showed that a catalyst containing <0.6 wt% Ru converted 5-HMF to 58% yield of DMF in propanol. In parallel, Lei et al. (2014) obtained 95% yield of DMF (in THF; 200°C in 2 h) while the direct hydrogenation of carbohydrate-derived HMF into DMF was also achieved, with DMF separation from the reaction mixture by distillation (Lei et al., 2014).

Other studies focused on high yields and selectivity toward DMF using “classical” mono and also bimetallic catalysts, including Pd and Ru (Hansen et al., 2012; Nishimura et al., 2014; Zu et al., 2014; Luo et al., 2015; Mitra et al., 2015) as well as less conventional catalysts (Gawade et al., 2016), which include biologically-derived materials. A preliminary study using cells of *Bacillus benzeovorans* as the catalyst support noted that, while classical 5 wt% Pd on carbon catalysts achieved 95% conversion of commercial 5-HMF (yield was 25% DMF in formic acid/trimethylamine), a bimetallic bio-catalyst of 2.5wt%Pd/2.5wt%Ru achieved 97% conversion with 50% selectivity (Omajali, 2015). In propanol the respective DMF yields (at 94% conversion) for the chemical Pd/C and bio-Pd/Ru were 33 and 42%, respectively (Omajali, 2015) but detailed studies using THF as the solvent were not undertaken. As far as the authors are aware, most studies have focused on up-conversion of commercially-obtained 5-HMF whereas this study focuses on 5-HMF within the product mix obtained from starch/cellulose thermochemical hydrolysis. A single stage reaction hydrolysis and up-conversion reaction formed the second aim of the study.

A commissioned consultancy report (Catalytic Management Technology Ltd., unpublished) noted that for a new catalyst to achieve market acceptance it must be better than commercially available comparators, or be cheaper to produce. For the latter, biogenic catalyst can be biorefined from metallic wastes into active neo-catalysts (Yong et al., 2010; Murray et al., 2017, 2019). However, growing cultures of bacteria solely for this purpose lowers the cost-effectiveness, and using “second life” bacterial cells left over from another biotechnology process has therefore been used to make active bio-metallic catalyst for a fuel cell (Orozco et al., 2010) and as a hydrogenation catalyst (Zhu et al., 2016). Therefore, the third aim of the study was to evaluate the potential using a consortium of acidophilic, sulfidogenic (CAS) bacteria left over from an unrelated biotechnology process for its ability to make bio-Pd/Ru catalyst for upgrading of 5-HMF, and to compare this with using a pure culture of the sulfate-reducing bacterium (SRB) *Desulfovibrio desulfuricans*, which was purpose-grown for the application.

The primary biotechnology process for the CAS uses H_2_S generated in a low pH sulfidogenic bioreactor to selectively remove and to recover metal resources (as sulfide precipitates) from metal-rich mine water wastes (N̆ancucheo and Johnson, 2012; Santos and Johnson, 2017, 2018). The bioreactors are operated in continuous flow mode and generate an effluent liquor that contains both bacterial cells and some residual sulfide A full-scale system has been estimated to generate several hundred liters of waste liquor/day (Murray et al., 2019). The bacteria, like *D. desulfuricans*, would contain residual biogenic sulfide, usually considered as a potent catalyst poison (Dunleavy, 2006). Against this, classical sulfidogenic *Desulfovibrio* (washed) cells produced a bio-Pd(0) catalyst that was as effective as that made by (non-sulfidogenic) *E. coli* (Deplanche et al., 2014) and also produced a better fuel cell electrocatalyst (Yong et al., 2007). A recent study has highlighted the role of palladium sulfide modifier to a Pd catalyst which was superior in the semi hydrogenation of alkynes (Albani et al., 2018). Analysis of bio-Pd(0) on *D. desulfuricans* confirmed the presence of sulfur by energy dispersive X-ray microanalysis, while the outermost ∼10 nm layer of washed cells was shown to comprise 1.3% atomic concentration of sulfur as determined by X-ray photoelectron spectroscopy (Omajali, 2015); the binding energy (eV) of peak positions for S2p was shifted from 165.39 to the lower binding energy of 163.97 after addition of PdII) (Omajali, 2015), which suggests the formation of a Pd-S bond (Gotterbarm et al., 2012). Hence this study sought to compare bio-Pd/Ru from the two types of sulfidogenic culture, placing the findings in the context of what is known about the 5-HMF upgrading reaction and the potential for “in process” energy generation within an integrated biorefinery and in the context of current developments in “classical” hydrogenation catalysts.

## Materials and Methods

### Thermo-Hydrolysis Reactions

The method for thermal hydrolysis was as described previously (Orozco et al., 2012). The batch reactor system for starch/cellulose hydrolysis comprised a bench top reactor (100 mL; Parr series 4590 pressure; maximum operating conditions: 200 bar; 350°C) of Type 316 Stainless Steel equipped with a heat/agitation controller **(**Parr 4848). All chemicals in the study were analytical grade from Sigma-Aldrich (potato starch, cellulose powder, 5-HMF and 2 methyl-tetra-hydro-furan.

For hydrolysis, starch (7.2 g) or cellulose (5.1 g) was suspended in de-ionized water (final reactant volume of 60 mL for starch; 120 g/L and 70 ml for cellulose; 72.9 g/L or as otherwise stated) and charged into the reactor for hydrolysis. The reactor was sealed and purged with N_2_ three times before pressurizing to 30 bar (N_2_) and heating to the set-point temperature (220°C for starch; 260° for cellulose) with agitation (300 rpm). Reaction conditions were held for 15 min before cooling to 35°C by submersion in cold water. The hydrolyzate was separated (after depressurization) from the solid residue (vacuum filtration; filter paper Fisherbrand QL100) or by centrifugation (10,000 rpm; 10 min). The reactions were repeated as required and pooled to produce sufficient quantities of starch and cellulose-derived 5-HMF. Hydrolyzates were kept at 4°C before analysis using a GC (Shimadzu 2010 with an autosampler AOC-20S, a FID detector and ZB-Wax column (30 m × 0.25 mm × 0.25 μm); injection volume 1 μL; inlet temperature 260°C; injector temperature 300°C; detector temperature; 300°C, inlet pressure 100 KPa; split ratio of 100:1 with H_2_ carrier gas at a flow rate of 1 mL/min). The heating regime was 0 min GC temp 100°C; 10 min GC temp 200°C; 22 min GC temp 200°C and 25 min GC temp 250°C. Reaction solid residues were not quantified nor analyzed.

### Solvent Extraction of 5-HMF

The method for 5-HMF extraction was based on the experimental determination of partition coefficients at batch and continuous conditions according to Blumenthal et al. (2016). In this study the mass transfer of 5-HMF from the aqueous to the organic phase was faster at 60°C and concentrations of 5-HMF in the range between 1 and 5wt% in the aqueous feed had little effect on the partition coefficients (not shown). The produced starch and cellulose hydrolyzates, respectively, were mixed in equal volumetric proportions with 2-MTHF (organic extraction solvent) in an Erlenmeyer flask [magnetic stirrer, 200 rpm, 60°C (temperature-controlled water bath); 20 min]. After extraction aqueous and organic phases were separated into: the top organic phase “supernatant” and the bottom aqueous phase “filtrate.” Both phases were sampled and kept at −20°C before analysis by GC. Solvent extraction efficiency was calculated according to Equation (1):

(1) $$\mathrm{Extraction efficiency}\;(\%)=\frac{\mathrm{moles of HMF in supernatant}}{\mathrm{moles of HMF in hydrolysate}}*100$$

### Catalyst Preparation

#### Bacterial Cultures

*Desulfovibrio desulfuricans* NCIMB 8307 was grown sulfidogenically as described previously (Omajali et al., 2015). Following harvest and washing (9,000 × *g*; 4°C; washed three times in 20 mM MOPS-NaOH buffer, pH 7.0) the cells were left at 4°C under nitrogen until use. The consortium of acidophilic sulfidogenic (CAS) bacteria (waste culture) was taken from a continuous metal waste treatment process with the H_2_S off-gas diverted into metal sulfide recovery from minewater. Two batches of CAS ∼15 L each, were collected independently over several days, harvested, washed as for *D. desulfuricans* and stored as a concentrated suspension at 4°C under air, routinely overnight, before metallization. Using terminal restriction enzyme fragment length polymorphism (T-RFLP) analysis, as previously reported for this microbial consortium (Santos and Johnson, 2017), the CAS was found to comprise 66% *Desulfosporosinus acididurans* (Sánchez-Andrea et al., 2015) 7% Firmicute strain CEB3, 10% *Acidocella aromatica* strain PFBC, 10% *Actinobacterium* AR3, and 7% *Acidithiobacillus ferrooxidans*. Cells were harvested and washed as for *D. desulfuricans* and left under N_2_ before metallization.

#### Preparation of Monometallic and Bimetallic Bionanoparticles

Commercial metal salts (NaPdCl_4_ and RuCl_3_) were from Sigma-Aldrich, as were 5wt% Pd and 5wt% Ru on carbon catalysts and commercial 5-HMF (≥99%) and 2,5-DMF (99%). For monometallic bio-Ru catalysts cell suspensions were suspended in 1 mM Ru (III) (RuCl3.2H_2_O solution; pH 2, in 10 mM HNO3) to the required biomass/metal ratio for the desired loading (wt%) and left for 30 min (30°C) for metal uptake by the cells. H_2_ was bubbled for ∼1 h through the Ru(III)-cells suspension then was left under H_2_ (sealed bottle; 180 rpm agitation; 30°C) for 96 h, with residual Ru(III) in solution analyzed by the tin chloride method (Charlot, 1978; Deplanche et al., 2010) to estimate the actual wt% loading on the cells (all of the Pd was removed onto the cells in the first step).

Synthesis of bimetallic Pd/Ru used, sequentially, a 2 mM Pd (II) and a 1 mM Ru (III) solution by the method of Deplanche et al. (2012) with modifications: 2 mM Pd (II) solution was reduced to Pd(0) on the cells under H_2_ (30 min; complete removal (by assay) of residual soluble Pd(II)) to give 5wt% bio-Pd. The bio-Pd was washed twice (distilled water) and then added to the required volume of 1 mM RuCl_3_ solution to give a final loading of (nominally) 5 wt% Pd/5wt% Ru. The Bio-Pd/Ru mixture was left to stand (1 h) then saturated with H_2_ (as above; 180 rpm agitation, 30°C; 96 h). Residual Ru(III) was estimated by assay (above). The presumptive bimetallic bio-NPs were washed three times (distilled water) and once with acetone (9,000 × g, 15 min, 4°C) air- dried and ground manually in a pestle and mortar.

### Scanning Electron Microscopy (SEM), High Resolution Scanning-Transmission Electron Microscopy (STEM) With HAADF (High-Angle Annular Dark Field) Detector, Energy Dispersive X-Ray Analysis (EDX), and Determination of Lattice Spacing

For STEM samples were fixed in glutaraldehyde [2 h; 4°C; 2.5% (w/v) in 0.1 M cacodylate buffer, pH 7.2], and, after washing (three times with the cacodylate buffer), were stained (1% aq. osmium tetraoxide). Thin sections were prepared for TEM as described previously (Deplanche et al., 2012), and electron opaque regions were examined by STEM and EDX using a FEI image Cs-corrector configuration Titan^TM^ G2 60–300 STEM microscope equipped with HAADF detector (accelerating voltage of 300 kV), with lattice spacings determined using “ImageJ” through profiling of high resolution HAADF-STEM images. For examination of the CAS mixed population by scanning electron microscopy (SEM) samples were mounted on aluminum stubs using carbon adhesive tape and coated with carbon (EMITECH K975X coater). The coated samples were observed using a Quanta 400 FEIESEM operating at an accelerating voltage of 5 kV.

### X-Ray Photoelectron Spectroscopy (XPS) of Material at Cell Surfaces

A few mg of samples were air-dried. XPS was used for analysis of surface chemical composition and determination of metal oxidation state (Kratos Axis Ultra DLD spectrometer; Kratos Analytical), as described by Omajali (2015), at room temperature. Illumination of samples used an Al Kα x-ray source, with emitted photoelectrons collected using a hemispherical electron analyzer. Survey spectra were acquired at a pass energy of 160 eV (resolution ∼2.0 eV), with the pass energy being reduced to 20 eV (resolution 0.4 eV) for the acquisition of high resolution core level spectra. As the samples were insulating, a charge neutralizer was used to prevent surface charging with a low energy electron beam directed on to the sample during XPS data acquisition. A take-off angle of 90° was used, to probe a depth of ∼5–10 nm to examine bio-NPs located at the outermost cell surfaces. Generated data were converted into VAMAS format and analyzed (CasaXPS package: Fairley, 2013) employing Shirley backgrounds, mixed Gaussian-Lorentzian (Voigt) line-shapes and asymmetry parameters where appropriate. All binding energies were calibrated to the C 1s peak originating from C-H or C-C groups at 284.8 eV. References were commercial 5 wt% Pd on carbon and commercial RuCl_3_.

### Synchrotron-Based Radiation-Scanning X-Ray Microscopy (SRSXM) Study of Elemental Pd, Ru, and Light Elements in Bio-Pd/Ru

Samples prepared as for TEM, thin-sectioned (0.25 μm) using a diamond knife on a Reichert Ultracut S ultramicrotome and stained as above, were examined using scanning X-ray microscopy [beamline IO8, Diamond Light Source (United Kingdom)^1^^,^^2^ ] typically operating at 3GeV energy of the storage ring with top-up injection mode at 300 mA current. The IO8 beamline at Diamond uses radiation in the 0.25–4.4 keV photon energy range, generated by an Apple II-type-undulator. X-ray fluorescence (XRF) elemental mapping data were acquired for the light elements using the K absorption edges and acquired from L edges for the metallic elements. XRF data analysis was performed using the PyMCA (Phyton Multichannel Analyser) software, a multiplatform code for the analysis of the ED-XRF spectra.

### Catalytic Upgrading of 5-HMF to 2,5-DMF

The catalytic transfer hydrogenation reactions were carried out in a stainless steel Parr reactor series 4590 as described above. Three sets of experiments were carried out: set 1 (commercially obtained 5-HMF); set 2 (starch-derived 5-HMF) and set 3 (cellulose derived 5-HMF) each using *D. desulfuricans* and CAS bio-catalysts. For set 1 the reactor was charged with 250 mg of commercial 5-HMF in 25 mL of MTHF (80 mM 5-HMF solution); sets 2 and 3 used appropriate volumes of 5-HMF in MTHF extracted from starch and cellulose hydrolyzates, respectively, to the same final concentration of 5-HMF. In all sets a weight ratio of 5-HMF:catalyst of 2.5:1 was added to the reactor. The catalysts tested were commercial Ru-C (5wt% Ru on charcoal: Johnson Matthey), biorecovered Ru (bio-Ru; 5wt% on CAS) and bimetallic preparations: 5wt% Pd/5wt% Ru bio-Pd/Ru (nominally) on *D. desulfuricans* and CAS cells. The reactor was sealed, purged 3 times with H_2_ (50 bar), pressurized with H_2_ (50 bar) and heated (260°C; 2 h; 500 rpm). After the reaction, the reactor was quenched to 35–40°C in a water bath and the reaction mixture was filtered (Fisherbrand QL100 filter paper). Samples were stored at −20°C before analysis.

### Analysis of Residual 5-HMF, 2,5 DMF and Co-products in the Catalytic Conversion Reaction

Samples were analyzed using a GC-FID for quantification and a GCMS-QP2010s for compound identification. All GC-FID analysis was performed on a Shimadzu GC2014 equipped with a Shimadzu AOC-20i autosampler. The carrier gas was hydrogen, supplied by an external hydrogen generator (Parker). The GC was fitted with a Restek Stabilwax-DA column (30 m length, 0.32 mm ID, and 0.25 μm film thickness). The injection volume was 1 μL with a 39 split ratio. The inlet temperature was 250°C. The detector was a flame ionization detector (FID) with a flame temperature of 300°C, and a sampling rate of 40 ms. The heating profile was 60°C for 2 min then heated to 200°C at 5°C/min followed by further heating to 240°C at 15°C/min where it remained for a further 3 min. Analysis was carried out using Shimadzu GC solutions software. Calibration curves were third order between 80 and 0.4 mM.

All GC-MS analysis was performed on a Shimadzu GCMS-QP2010s equipped with a Shimadzu AOC-20i autosampler. The carrier gas was helium. The GC was fitted with a Restek Rxi-1ms column (15 m length, 0.25 mm ID, and 0.25 μm film thickness). The injection volume was 1 μL with a -1 split ratio. The inlet temperature was 250°C. The detector was a single quadrupole mass spectrometer in electron ionization mode. The detector and interface temperatures were 250°C. The detector acquisition mode was scanning between 40 and 400 m/z, with a scan every 300 ms. The solvent cut time was 1 min. The heating profile was 60°C for 2 min then heated to 200°C at 5°C/min followed by further heating to 240°C at 15°C/min where it remained for a further 3 min. Analysis was carried out using Shimadzu GCMS Real Time Analysis and Shimadzu GCMS Post Run Analysis software. 5-HMF conversions and 2,5 DMF yields were calculated as follows (Equations 2–5):

(2) $$\mathrm{HMF conversion}\;(\%.\;\mathrm{SM: starting material})=1-\left(\frac{\mathrm{moles of HMF in products}}{\mathrm{Starting moles HMF}}\right)*100$$

(3) $$\mathrm{DMF yiels}\;(\%)=\frac{\mathrm{moles of DMF in products}}{\mathrm{Starting moles HMF}}*100$$

(4) $$2,\;5\;\mathrm{DMF selectivity}\;(\%)=\left(1-\frac{\mathrm{moles of}\;2,\;5-\mathrm{DMF in products}}{\mathrm{starting moles of}\;5-\mathrm{HMF}-\mathrm{final moles of}\;5-\mathrm{HMF}}\right)\times 100$$

(5) $$\mathrm{DMF energy}\;\left(\frac{\mathrm{MJ}}{\mathrm{kg SM}}\right)=\left(\frac{\mathrm{kg of DMF in products}}{\mathrm{kg SM}}\right)*\left(\frac{\mathrm{MJ}}{\mathrm{kg DMF}}\right)$$

## Results and Discussion

### Conversion of Starch and Cellulose to 5-HMF and HMF-MTHF Extraction

The yields of 5-HMF from thermal hydrolysis of starch and cellulose were 130 and 40 mg 5-HMF/g starting material (aq.), respectively. MTHF extraction efficiency of 5-HMF from the aqueous phase was between 59 and 63% (from several preparations) resulting in 5-HMF concentrations in MTHF of 70 and 21 mM for starch and cellulose, respectively, when pooled (Table 1). Example extraction efficiencies from two independent preparations were 59.3 and 62.8%, however, the actual efficiency is of relatively low importance for proof of principle since this specific method would require further development for scale up and final application. For example a cost-benefit analysis would consider a raft of solvents for efficiency and economy at scale although the potential benefirts of MTHF are already apparent (see Introduction).

**Table 1:** Conversion of starch and cellulose to 5-HMF and extraction efficiency of MTFH.

	Starch	Cellulose
Conversion to 5-HMF (mg 5-HMF/g SM^∗^)	130	40
MTHF extraction efficiency of 5-HMF (%)	59.6	62.8
[5-HMF] in MTHF (supernatant; mM)	70	21

*^∗^SM = starting material (starch or cellulose).*

The yield of 5-HMF obtained from starch hydrolysis was consistent with previous work (Miyazawa, 2005; Orozco, 2012) but the yield from cellulose was markedly lower (Table 1). This could be expected as the crystalline structure of cellulose makes it more difficult to hydrolyze, requiring higher temperatures, however, this also enhances reaction degradation pathways of 5-HMF to simpler structures such as formic and levulinic acids (Minowa et al., 1998; Palmqvist and Hahn-Hägerdal, 2000). This additional complexity was confirmed by examination of example product mixtures by GC (Supplementary Figure S1A) but the actual amounts of products (other than DMF) were not determined.

The yield from cellulose was ∼2.5 times lower than values reported from hot compressed water hydrolysis of cellulose (e.g., ∼110 mg 5-HMF/g cellulose @ 260°C) (Minowa et al., 1998; Kamio et al., 2006; Orozco, 2012). Minowa et al. (1998) studied the decomposition of cellulose in hot compressed water with alkali or nickel catalysts; the positive effect of these on the cellulose degradation pathway to products was significant as compared to catalyst-unsupplemented conditions. The reactor vessel used by Kamio et al. (2006) and Orozco (2012) was made of Hastelloy C-22 and C-276, respectively, both containing a nickel (∼ 55%)-molybdenum-chromium alloy. Unpublished work (Orozco, 2012) attributed an improvement in cellulose conversion to possible leaching of catalytic metals from the reactor body. These materials, when corroded, are known to leach metals to the reaction medium thereby possibly affecting cellulose decomposition and product distribution (Yu et al., 1993; Lu et al., 2006). Possible benefits of catalyst-enhanced hydrolysis may be suggested, however, the presence of heavy metals in the hydrolyzate is likely to inhibit its downstream fermentation due to metal toxicity. The use of added catalyst (and its removal from the product mixture) was beyond the scope of this study although immobilized biofilm-catalyst has been used in other applications, e.g., of removal of toxic Cr(VI) via bio-Pd-mediated reduction (Yong et al., 2015). The reactor used in the present work comprised stainless steel 316 containing Fe (∼65%), Ni (∼12%), and Cr (17%) alloy; metal leaching was not determined but successful biohydrogen fermentations of the hydrolyzate were reported following removal of toxic 5-HMF (Orozco, 2012). The lower yields of 5-HMF and the presence of more degradation products in the cellulose hydrolysate (Supplementary Figure S1A) would have an adverse effect on the delivery of additional energy. Ideally the continuous extraction of 5-HMF during the biomass hydrolysis reaction would be desirable to avoid further degradation of this compound which occurs at high temperatures (Saha and Abu-Omar, 2014), e.g., by addition of a solvent extraction loop with organic phase removal. This would be particularly important for 5-HMF derived from hydrolysis of cellulose, which typically requires a higher reaction temperature.

The MTHF extraction efficiency of 5-HMF from the aqueous phase was comparable, at ∼60 and 63% from starch and cellulose hydrolyzates, respectively, delivering organic supernatants containing 8.8 and 2.2 g/l (70 and 21 mM) of 5-HMF (Table 1). The initial concentrations of 5-HMF in the aqueous phase and the effect of sugars present in the hydrolyzate will have an influence on the partition coefficient of 5-HMF which can be significantly higher with increasing concentrations. For example, the presence of 30% wt/vol fructose in MTHF increased the partition coefficient of 5-HMF from 2.1 up to 36% (Blumenthal et al., 2016). The goal is separation and catalytic upgrading to DMF in the same solvent, hence the 5-HMF partition coefficient must be maximized while minimizing the glucose partition coefficient. These studies were beyond the scope of this investigation, however, a lack of solubility of glucose in MTHF suggests it would be unlikely that glucose was removed from the hydrolyzate during the 5-HMF extraction process.

Other approaches for 5-HMF removal such as over-liming, activated carbon, zeolites and ion exchange resins selectively removed up to 85% of 5-HMF (Ezeji et al., 2007; Hodge et al., 2009; Jin et al., 2015; Ma et al., 2017). However, the recovery of 5-HMF from these extraction substrates is either difficult or unfavorable for downstream processing into 2,5-DMF due to high intramolecular attraction between 5-HMF and the extraction substrates or the regenerant/eluting agents containing water or water miscible organic solvents (Liu et al., 2015).

The efficiency of MTFH in the hydrogenation reactions compared to THF was evaluated in a set of reactions as shown in Supplementary Table S1A. In all cases MTHF proved to be a better solvent than THF, delivering higher conversion and yields of DMF. It is concluded that MTHF is able to both extract 5-HMF from the hydrolyzate and serve as the solvent for its upgrading, facilitating a one-stage reaction.

### Hydrogenation of 5-HMF Into 2,5 DMF Using Ru and Pd Catalysts

The Pd was removed onto the bacteria in the first step to give 5wt% loading of Pd. In the second step all of the Ru(III) was removed by the CAS cells (loading was 5wt%Pd/5 wt% Ru) whereas the *D. desulfuricans* only loaded Ru (III) to 3 wt% (actual catalyst composition was 5%Pd/3%Ru). The reason for this was not investigated further (see later Discussion).

**FIGURE 1:**
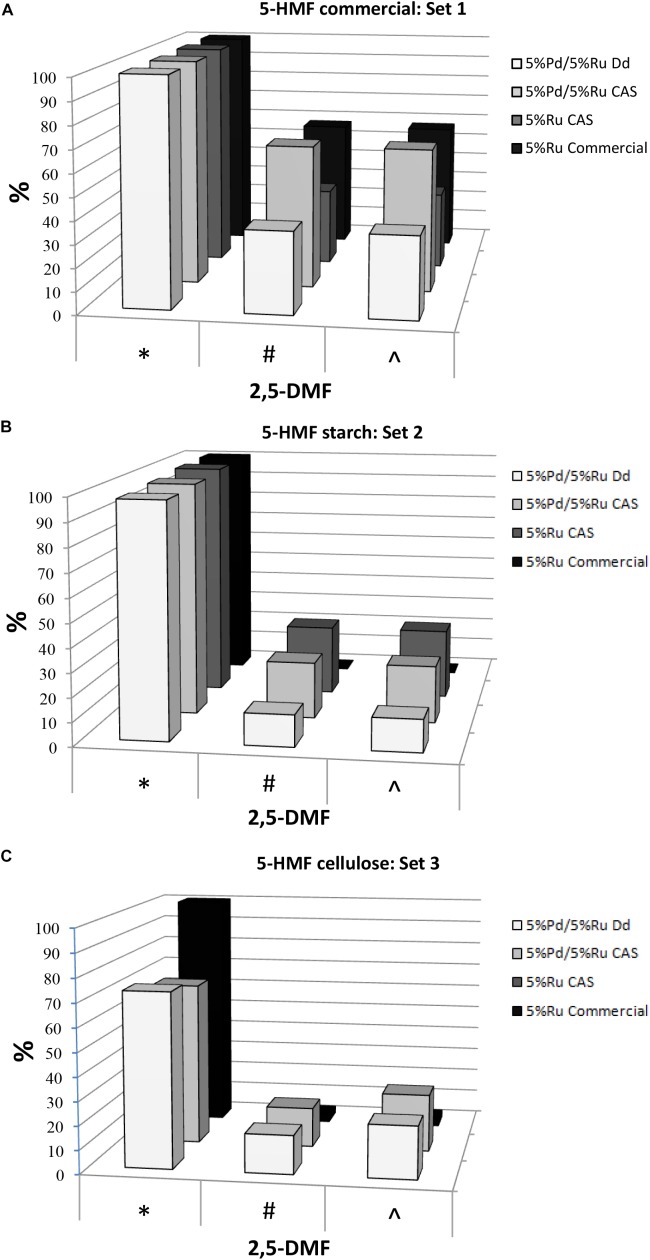
Conversion of 5-HMF from commercial source **(A)** and from starch **(B)** and cellulose **(C)** hydrolyzates by commercial 5%Ru on carbon catalyst, 5wt% bio-Ru on sulfidogenic waste culture (CAS), 5wt%Pd/3wt%Ru on *D. desulfuricans* (nominal loading was 5wt% Pd/5wt% Ru: see later) and 5wt%Pd/5wt%Ru on CAS as shown. Experiments were done at least twice on separate occasions and data are shown in Supplementary Table S1B. X axis: ^∗^Conversion of 5-HMF (%). ^#^Yield of 2,5-DMF (%). ^∧^Selectivity to 2,5-DMF (%).

The hydrogenation tests for the three 5-HMF substrates are summarized in Figure 1 (with the data and errors shown in Supplementary Table S1B) For set 1 (pure 5-HMF) and set 2 (5-HMF from starch) the conversion of 5-HMF to products was generally between 95 and 100% (Figure 1). In contrast using 5-HMF from cellulose (set 3) the conversion was between 61 and 81% reaching 100% only with 5%Ru commercial catalyst but delivering a very poor yield of DMF (3%: Figure 1C). It is concluded that, while commercial Ru catalyst is useful for DMF production from pure 5-HMF, it is virtually ineffective in producing DMF from the hydrolyzates (Figure 1B,C). The reason for this was not investigated but may be due to fouling of the commercial catalyst by reaction components or products or to over-reaction yielding other products which were neither identified nor quantified. Example chromatograms showing reaction products are shown in Supplementary Figure S1B. Notably, both types of bio-Pd/Ru gave significant DMF product from the hydrolyzates where the commercial counterpart was ineffective (Figure 1A–C and Supplementary Table S1B). In addition, using cellulose hydrolyzate, both types of bio-Pd/Ru bimetallic performed comparably (Figure 1C) whereas the DMF yield and selectivity in starch hydrolyzate using CAS was ∼ double that yielded via catalyst made using bio-Pd/Ru on *D. desulfuricans* (Figure 1B). This difference was not attributable to an effect of any component of the hydrolyzate since the effect was clear also with pure 5-HMF substrate (Figure 1A).

The highest yields of DMF obtained corresponded to 5%Pd/5%Ru on CAS using commercial 5-HMF (63.13%) and 5% Ru on CAS cells using 5-HMF from starch (29.3%) this being ∼46% lower; in both cases almost 100% 5-HMF conversion was achieved. This difference using the starch hydrolyzate could be attributed to the occurrence of side reactions caused by the presence of other by products in the starch derived 5-HMF (see GC chromatograms; Supplementary Figure S1B). The commercial Ru-catalyst gave 57% yield of DMF using pure 5-HMF (set 1) but when reacted on starch-derived 5-HMF (set 2) the yield was negligible which has negative implications for the application of the commercial catalyst in biomass product upgrading.

In terms of potential energy to be gained from the produced DMF: set 2 (5%Pd/5%Ru CAS) would give 2.1 kWh/kg starch-derived 5-HMF and set 1 (5%Pd/5%Ru CAS) 4.6 kWh/kg commercial 5-HMF. These energy yields would contribute to mitigate by ∼ 28 and 63%, respectively, of the “parasitic” energy needed for the hydrolysis and catalytic reactions (7.3 kWh/kg 5-HMF) assuming 80% heat recovery from the reactions. The equivalent potential energy gains using the bimetallic catalyst made on *D. desulfuricans* would be 0.95 kWh/kg from starch-derived 5-HMF. The >2-fold better performance of the catalyst made on the CAS as compared to *D. desulfuricans* prompted comparison of metal deposition by the two sulfidogenic cultures. As noted above, the *D. desulfuricans* loaded 60% of the Ru(III) and hence the approximate proportions of metals (Ru:Pd) were 0.6:1 and 1:1, respectively, for the *D. desulfuricans* and CAS materials; other possible differences between them were sought.

### Formation of Bimetallic Material by Washed Cells of the Sulfidogenic Cultures

#### Bio-Pd/Ru Supported on *D. desulfuricans*

Palladium (II) was completely removed from the challenge solution by both sets of cells (estimated as in Omajali et al., 2015) and uptake into/into the cells to 5wt% Pd was concluded. Formation of Pd(0) on *D. desulfuricans* was described previously (Omajali et al., 2015). Here, at a loading of 20 wt% Pd both cell surface-localized and intracellular Pd-nanoparticles were observed (Supplementary Figure S2). The occurrence of metal intracellularly implies an uptake mechanism but its relatively low occurrence in the cytoplasm could imply an effective efflux mechanism as a detoxification response which is a well known metal resistance mechanism and a way to ensure that cells retain essential metals while rejecting toxic ones (Waldron and Robinson, 2009). At a loading of 5 wt% Pd (i.e., for the Pd pre-loading as used in this study) little intracellular Pd(0) was observed (Supplementary Figure S3 and inset). This is in contrast to (non-sulfidogenic) *E. coli* where Pd-NPs were visible throughout the cells (Supplementary Figure S3 and inset). While Pd deposition in *E. coli* did not co-map with either phosphorus or sulfur the elemental maps (Supplementary Figure S3) indicated a co-deposition of Pd with S, at least in part, in the cell surface layers of washed cells of *D. desulfuricans*. This is in accordance with earlier data from X-ray photoelectron spectroscopy that indicated formation of Pd-S bonds (and hence some palladium sulfide species) in the cell surface (outermost ∼ 10–30 nm) layers of *D. desulfuricans* (Omajali, 2015). It is not known if the putative palladium sulfide was formed from incoming or effluxing Pd(II).

**Table 2:** The sulfidogenic waste culture used in the study compared to *D. desulfuricans*.

Bacterium	% representation	Gram stain	Sporeformer
*D. desulfuricans*	100	Gram negative	−
*CAS: Desulfosporosinus acididurans^∗^*	66	Gram positive	+
CAS: Unidentified strain CEB	7	NK	NK
CAS: *Acidocella aromatica*	10	Gram negative	−
CAS: *Actinobacterium*	10	Gram positive	+
CAS: *Acidithiobacillus ferroroxidans*	7	Gram negative	−

*NK, not known. Only 17% of the CAS population were the same cell type as D. desulfuricans (Gram negative, non-sporeformer). The majority were Gram positive sporeformers (76%).*

**Table 3:** Atomic percentages of outermost ∼10 nm of metallized bacterial cells determined by XPS.

Sample	Ru	Pd	C	0	N	S	Cl	P	Ca
*E. coli* 5%Pd/4.7%Ru^∗^	2.42	0.14	71.48	19.81	4.30	0	0	0.80	0
*D. desulfuricans* 5%Pd/3%Ru	1.03	0.12	68.88	21.44	5.18	0.38	0.17	0.38	0.21
CAS 5% Ru	1.40	0	64.37	21.95	7.08	1.18	0	0.41	0
CAS 5%Pd/5%Ru	5.30	0.41	64.36	18.78	6.01	3.55	0.11	1.39	0

*^∗^E. coli was included as a comparator Gram negative bacterium that is non-sulfidogenic (Gomez-Bolivar et al., in review). The low content of the Pd at the cell surface is attributable to internalization into the lower cellular layers beyond the detection of XPS.*

Previous studies (Omajali et al., 2015; Williams, 2015) identified three populations of palladium nanoparticles (NPs) in *D. desulfuricans*: NPs in the cell surface layers, in the cytoplasm and localized within nuclear bodies (NBs: the intracellular inclusion nuclear body is shown in Supplementary Figure S2). NBs are a condensed form of DNA that commonly occurs in cultures in stationary phase or when grown at a slow growth rate (Zaritsky et al., 2017). Pd is well known to bind to DNA (Pillai and Nandi, 1977) and/or histone-like, DNA-associated proteins. Actively growing cells produce H_2_S, and a growing culture comprises a mixture of “young” (freshly divided) and “old” cells as well as dead cells. Hence, the *D. desulfuricans* culture would comprise a population of cells each at different stages of their cell cycle, with actively metabolizing and also senescent cells, from which H_2_S would possibly not be produced from residual metabolism. The possibility that incoming Ru(III) faces a potential “choice” between Pd(0), PdS (or other localization foci other than Pd-“seeds”) has not been considered previously as sulfidogenic cultures have not been examined in detail (Omajali, 2015; Supplementary Figure S3).

**FIGURE 2:**
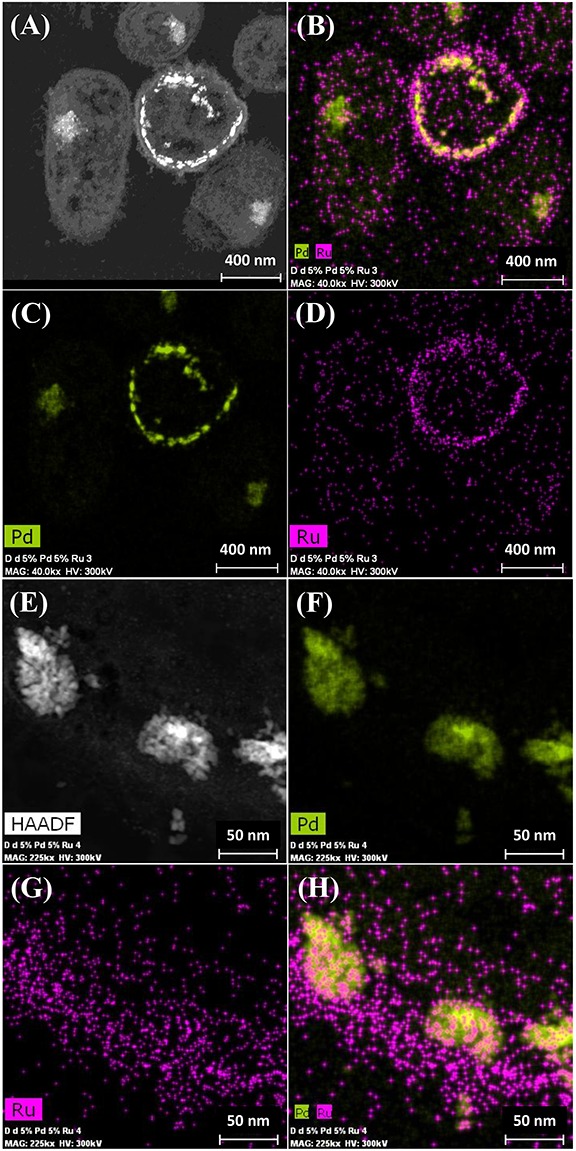
Deposition of 5wt% Pd/3wt% Ru by *Desulfovibrio desulfuricans* and co-localization of the metals on the cells. Arrows: dense nuclear bodies typical of slowly-growing cells. **(A)** HAADF image where metallic NPs appear bright. **(B)** elemental map of Pd (green) and Ru (magenta); individual elemental maps are shown in **(C,D)**. **(E)** HAADF image of discrete NPs in the cell surface region mapped for Pd (**F**, green) and Ru (**G**, magenta) and co-localization of Pd and Ru **(H)**.

**FIGURE 3:**
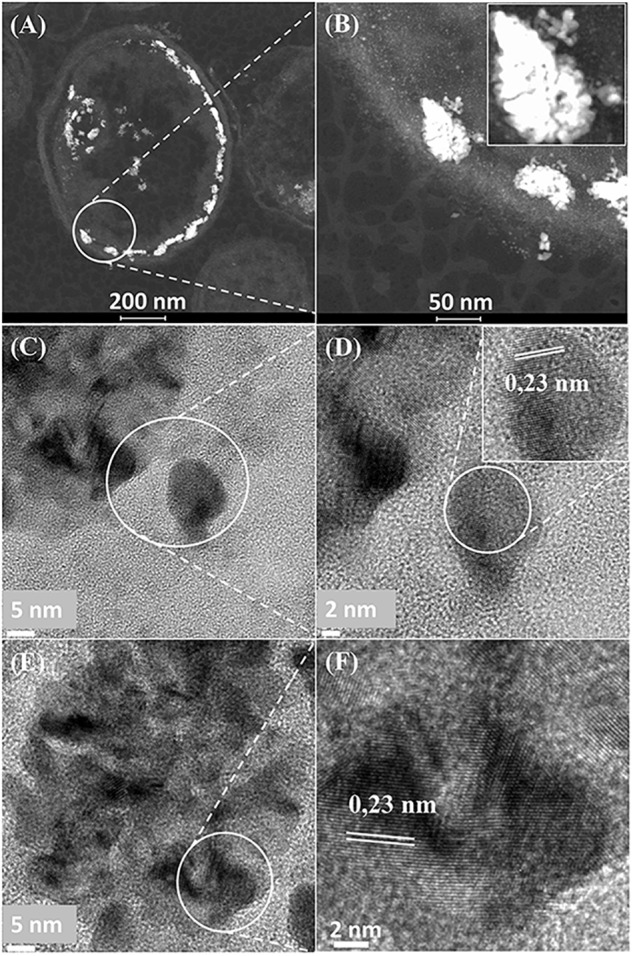
**(A)** Region of cell surface shown in Figure 2E–H. Enlarged image (**B**, inset) shows that the large NPs are agglomerations of smaller ones. Crystal lattice spacings are shown for a NP outside **(C,D)** and within **(E,F)** an agglomeration.

It was assumed that 5 wt% Pd(0) NPs serve as the putative “seeds.” for Ru deposition, on the basis of earlier work on the formation of Pd/Au bimetallic catalysts (Deplanche et al., 2012). However, unlike with Au(III) reported previously using *E. coli* (Deplanche et al., 2012), the Ru(III) was incompletely removed by *D. desulfuricans* (see above) and the metal loading onto the cells was 5wt% Pd/3wt% Ru. Figure 2A shows the formation of metallic NPs in the nuclear bodies of *D. desulfuricans* (Figure 2A,C) and also localized at the cell surface. The deposition of Ru in the cells was greater than the background, but there was no clear association with Pd or any cellular feature in cells showing nuclear bodies (Figure 2B,D) whereas a co-localization of Pd and Ru was evident in cell surface layers (Figure 2B and Supplementary Figure S4). Putative bimetallic structures occurred at the cell surface, in addition to localizations where an association between Pd and Ru (with apparent Ru outgrowths) was suggested (Supplementary Figure S4). In contrast, in another example cell, discrete Pd-NPs in the surface layers showed no clear association of Pd and Ru and deposition of the latter appeared to be uniform throughout the cell surface (Figure 2E–H). The distribution of Ru appeared to be independent of the Pd-NPs (Supplementary Figure S4) and it is not known whether the apparent Ru “overgrowths” were coincidental or in association with Pd nucleation. Examination of examples of cell surface regions showed (Figure 3A,C–F) the large NPs to comprise agglomerations of smaller ones of sizes ∼ 5–7 nm (Figure 3B, inset) with lattice fringes of 0.23 nm. This could correspond to the (110) plane of RuO_2_ (Soin et al., 2012) but the (111) plane of Pd(0) was noted by Divakar and Raghunathan (2003) as 0.24 ± 0.01 nm and so the bio-NPs could equally be reporting the (111) facets of Pd(0). Pd(0)-NPs on *D. desulfuricans* were reported with Pd (111) lattice spacings of 0.250, 0.258, and 0.243 nm (Omajali et al., 2015). However, recent work in a purely chemical system has noted lattice spacings of palladium sulfide as 0.250 and 0.256 nm, attributed to the expansion of the Pd(0) lattice by sulfur (Albani et al., 2018). Hence, the identity of the bio-nano crystals we report here cannot be attributed with certainty (see later Discussion).

#### Waste Culture of Consortium of Acidophilic Sulfidogens (CAS)

*D. desulfuricans* is a Gram negative anaerobic SRB that respires at the expense of sulfate in lieu of oxygen. The final product is H_2_S. Early work to remediate acid mine drainage waters (AMD) developed the use of acidophilic (acid-tolerant) sulfate-reducing bacteria (see Introduction) to precipitate heavy metals as their sulfides. Later work developed a mixed sulfate-reducing bacterial consortium into a continuous process whereby the H_2_S off-gas precipitated metal sulfides, leaving the bacterial cells as the waste for use in this study. Samples were taken from the culture (in two periods separated by several weeks) that had been operating in a continuous mode for >5 years.

Examination of the CAS culture using SEM showed a variety of cell types (Supplementary Figure S5A), mostly comprising rod-shaped cells, some round structures and some small round bodies (presumably spores) both free and budding from some of the cells. Examination of the cell surfaces (bulk population) by XPS (see later) showed no calcium; calcium dipicolinate (CDP) is a major component of the bacterial spore and hence it may be concluded that the occurrence of bacterial spores in the mixture was below the limit of detection. However, the CDP resides below the outermost spore coat, being released upon germination (De Vries, 2004) and, since the penetration depth of XPS is in the order of 10–30 nm, this is not a definitive conclusion but may tend to confirm the low occurrence of spore-type small round structures visible by SEM (Supplementary Figure S5A).

Analysis of the CAS cell population using molecular biology identification methods revealed its composition as 66% *Desulfosporosinus acididurans* (sp. *nova*: Sánchez-Andrea et al., 2015) 7% unidentified strain CEB, 10% *Acidocella aromatica* strain PFBC, 10% *Actinobacterium* AR3 and 7% *Acidithiobacillus ferrooxodans* (Santos and Johnson, unpublished; Table 2) Hence, although the *D. desulfuricans* and 17% of the mixed culture would be united by their Gram-negative stain and non-sporeforming characteristic, 76% of the CAS would comprise Gram positive sporeformers.

The Gram-negative bacterial cell surface comprises a phospholipid outer membrane (OM) containing lipopolysaccharide that often bridges into extracellular hydrated polymeric materials which collapse upon drying. Beneath the OM the periplasmic space comprises a hydrated gel-compartment of width ∼ 30 nm and beneath that the inner membrane (IM), which is the cellular permeability barrier bounding the cytoplasm. The periplasmic space contains structural peptidoglycan, the N-acetyl glucosamine components of which would provide amine groups for potential coordination of incoming metals. The classical Gram positive cell lacks the OM and periplasmic space and its single membrane is bounded externally by a thick layer of peptidoglycan (as in the Gram-negative periplasmic space), which also contains phosphate in the form of teichoic acids embedded within it. In addition, there is often a coat of regular protein structures, the “S-layer” (which can also be found on Gram-negative cells) which is present in many Gram positive bacteria including *Bacillus sphaericus* strains isolated from heavy metal-contaminated sites (Merroun et al., 2005). This outermost surface layer plays a major role in the coordination of heavy metals and radionuclides through its carboxyl and phosphate groups (Merroun et al., 2005). In addition, archaea and Gram positive bacterial S-layer has been used as a template for the fabrication of metallic NPs of Au (Merroun et al., 2007; Bartolome et al., 2012). Bacterial cell surfaces have been extensively reviewed *per se* and in the context of metal binding behavior (e.g., Beveridge, 1989). It is assumed that binding of Ru(III) to Gram positive cells accesses more, or different, nucleation sites than onto Gram negative cells (e.g., via the higher content of surface-accessible petidoglycan in the former) but this was not tested.

#### Formation of Metallic Nanoparticles on CAS

**FIGURE 4:**
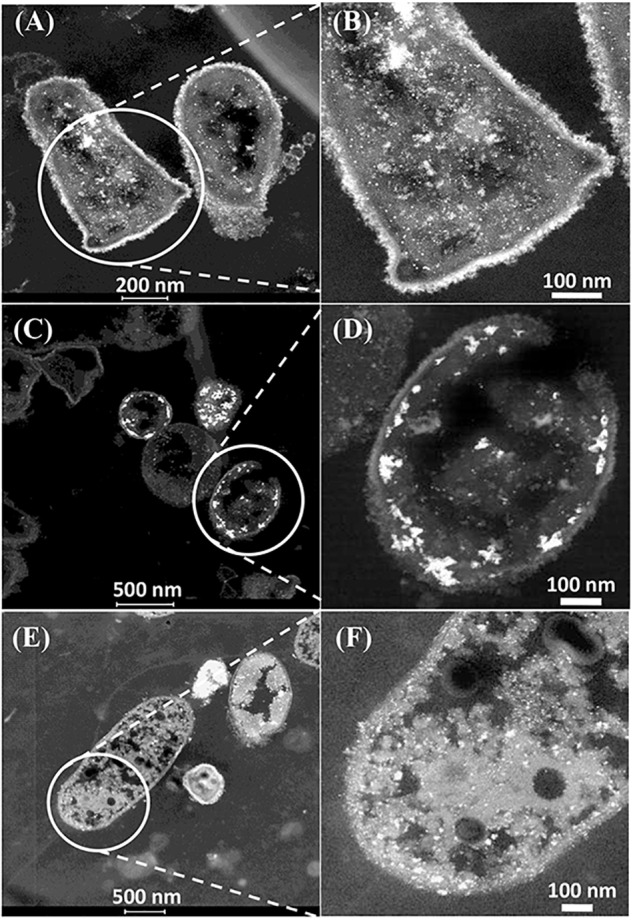
Examination of the CAS bacteria loaded with 5wt%Ru/5wt%Pd. Three main patterns of metal deposition are attributed to type I **(A,B)**, type II **(C,D),** and type III **(E,F)** cells.

The CAS was metallized with 5wt%Pd/5wt%Ru and examined in the light of the above discussions (Figure 4). Electron micrographs of the metallized CAS are also shown in Supplementary Figures S5B,C, S6. The cell heterogeneity is apparent in Supplementary Figure S5 and expansion of cell surface areas (Figure 4 and Supplementary Figure S6) shows four types on the basis of their pattern of metal deposition (c.f. Table 3) but each type could not be attributed to a taxonomic group on the basis of morphology alone. Type I cells (Figure 4A,B) showed heavy metallic deposits at the outer edge of the cell surface in addition to intracellular nanoparticles. Type II (Figure 4C,D) showed no outermost metal deposits, but instead dense metallic deposits co-localized beneath the wall layers and some intracellular NPs. Type III (Figure 4E,F) showed metallic NPs within the wall layers and also intracellularly. Figure 4E also shows a putative spore (type IV) but as the small round structures were numerically sparse (Supplementary Figure S5A) these were not considered to play a major role, although metallic deposits were apparent on the surface and within the putative spores (Supplementary Figure S6G). Some cells showed outer membrane vesicles (Figure 4A and Supplementary Figure S6H), too few in number to contribute to the overall metal deposition.

**FIGURE 5:**
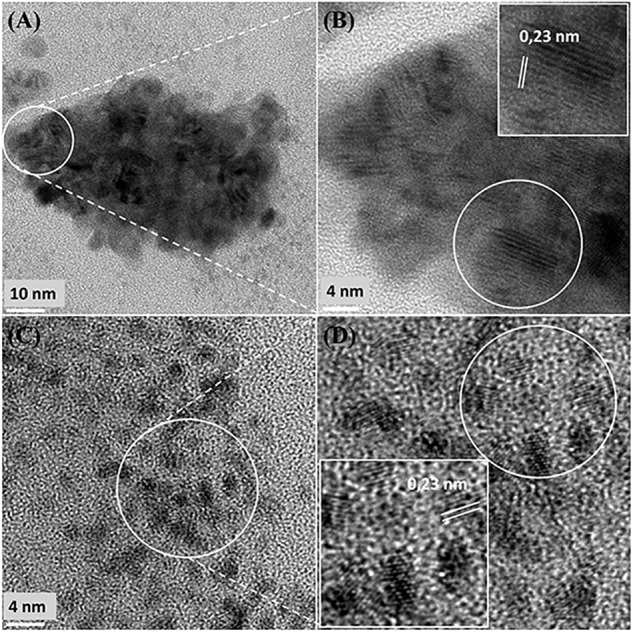
High resolution-TEM analysis of cell sections showing cluster **(A,B)** taken from Figure 4B (Panel B) and **(C,D)** taken from Figure 4B (Panel D) shows lattice spacing of 0.23 nm.

Further examination of the metallic NPs is shown in Supplementary Figure S6. Note that in type I cells (Supplementary Figures S6A,B) the NPs are generally separated, located on the outer face of the cell wall and of dimensions ∼ 2 nm whereas in type II cells (Supplementary Figures S6C,D) the NPs are larger (e.g., >50 nm) and appear as agglomerations. Type III cells (Supplementary Figures S6E,F) appear to have a layered cell surface structure (Supplementary Figure S6F) typical of a Gram negative cell but this was not confirmed. As with *D. desulfuricans* (above) the lattice fringes were 0.23 nm (Figure 5), i.e., the nanocrystals could have been either Pd(0) or RuO_2_ or, indeed, a form of palladium sulfide (see above). Much of the NP material appeared to be amorphous or non-crystalline as evidenced by a lack of lattice fringes.

**FIGURE 6:**
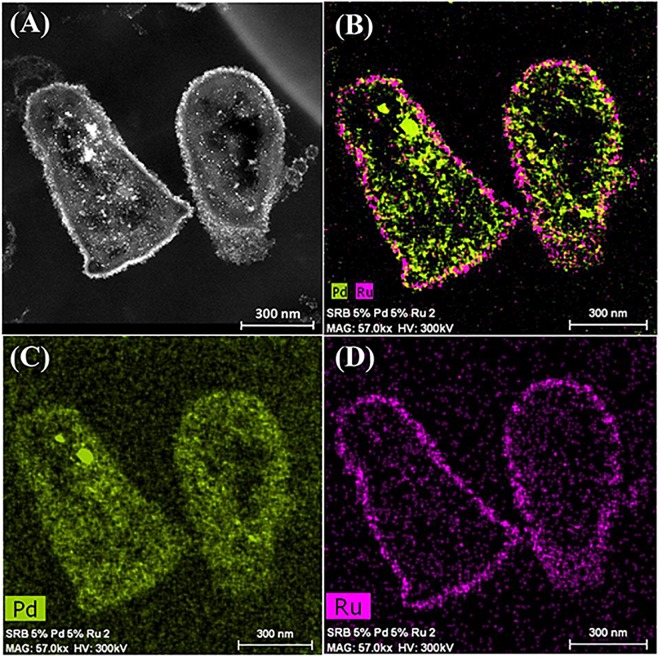
Elemental mapping of distribution of Ru and Pd in two type I cells (as in Figure 4) showing superimposition of Pd (green, **B**) and Ru (magenta, **D**) occurrences. Individual maps **(C,D)** show very little Ru inside the cells although Pd is distributed uniformly between the cell surface and intracellular regions.

Elemental mapping by energy dispersive X-ray microanalysis (Figure 6) showed that, in type I cells, while Pd was dispersed throughout the cell surface and cytoplasmic layers, the Ru deposits were almost exclusively confined to the cell surface with occasional intracellular occurrences scarcely higher than the background between the cells (Figure 6C,D). Examination of cell areas (Supplementary Figure S7) suggested that, where both elements occurred together, they tended to be co-localized, but areas of solely Pd-NPs were visible. The large Ru-NPs tended to occur as overgrowths onto Pd-NPs. While it is possible to assign a numerical correlation to the co-occurrence of specific elements in NPs (Omajali, 2015) this was not possible in the present study due to the small size and poor definition of the NPs, preventing estimation of NP boundaries (e.g., Supplementary Figure S7F).

**FIGURE 7:**
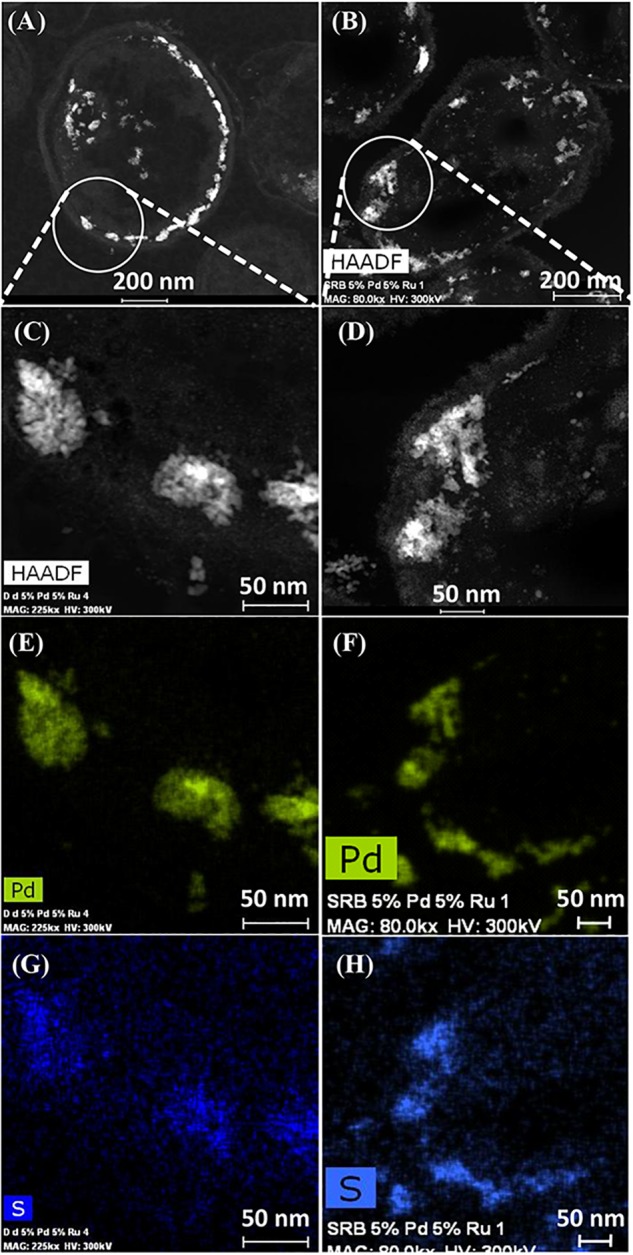
HAADF/STEM images of *D. desulfuricans* **(A)** loaded with 5wt% Pd/3wt% Ru and CAS cells type II loaded with 5%Pd/5%Ru **(B)**. Magnifications show similar clusters on sulfidogenic bacteria **(D)** and *D. desulfuricans* **(C)** located in the periplasm. Qualitative analysis using EDX elemental mapping shows superimposition in CAS bacteria for Pd **(F)** and sulfur **(H)** and also in *D. desulfuricans* **(E,G)**.

Type II cells in the CAS showed metallic deposits mainly at the cell surface in a similar way to that described for *D. desulfuricans* (see earlier). On the basis of the lattice fringes (see earlier) metal sulfides could not be discounted; even though the cells were washed a faint odor of H_2_S was detected in the CAS on standing after a few days. As no exogenous sulfate was provided this H_2_S may have arisen due to protein turnover; the presence of cellular storage materials to provide metabolic energy for turnover in the resting cells was not sought. However, given that the CAS was a mixed culture consortium evolved over 5 years, the possibility of cross-feeding may provide an evolutionary advantage under nutritionally sparse conditions, e.g., the presence of sulfide-oxidizing bacteria *Acidithiobacillus ferrooxidans*, which accounted for 7% of the culture (Table 2) would likely generate oxidized sulfur species for re-reduction into metal-accessible sulfides. These could arise from thiols arising from protein degradation, from cellular molecules such as glutathione or from histone proteins released from DNA during senescence. Alternatively (or in addition), given that polysulfides are now reported as cellular stores of sulfur in sulfide oxidizing bacteria and almost all of the sulfide in the reported case was oxidized to sulfate under low sulfide-flux conditions (Berg et al., 2014) there is a strong possibility that endogenous H_2_S may be generated from within the culture (via nascent SO_4_^2−^) under “resting” conditions via inter-species turnover. In addition, Newman et al. (1997) reported that *Desulfotomaculum* (a sulfate-reducer) precipitated arsenic trisulfide. The ability of bacteria to store sulfur is well recognized (e.g., Pickering et al., 2001; Pickering and George, 2002). In this case the storage material comprised globules of sulfur and not polysulfide (Pickering and George, 2002). Elemental mapping (Figure 7) showed co-localization of palladium and sulfur with a greater density of sulfur in the CAS sample as compared to *D. desulfuricans*, which had no access to additional oxidized forms of sulfur to reduce to thiols/H_2_S during NP formation. Although the EDX mapping method is qualitative only the result was confirmed in hydrated samples by complementary X-ray mapping (below). As far as the authors are aware, this is the first report of potential bacterial sulfur cycling being harnessed to the generation of novel catalytic biomaterials.

### Co-localization of Pd, Ru, and S Using X-Ray Mapping of Hydrated Specimens of CAS

Synchrotron radiation based scanning X-ray microscopy was used to determine the distribution of chemical elements (e.g., Pd, S) within the metallic NPs produced by hydrated specimens of CAS. XRF elemental mapping analysis was carried out at K and L edge of S and Pd, respectively. The results obtained (Supplementary Figure S7B) showed a close association of S and Pd at microscale (size of the analyzed region was 20 μm/70 μm). This microscale analysis is complemented by that of the EDX mapping method of the STEM/HAADF system. The major limitation of the EDX mapping method is that, while it is specific for the elements of interest (as long as their X-ray emission peaks are well separated) and can measure accurately most elements (but not the light elements like N, C) only a few cells can be examined within a field of view, albeit with mapping of specimen microareas within a single cell. In contrast the sensitivity of mapping X-ray emissions under illumination by synchrotron radiation is greater than from EDX and can co-map the light elements but this method has a limit of resolution of about 20 microns and hence it can best image cell clusters in a given field of view and provide a numerical analysis of “population” co-occurrence; this then takes into account the presence of different cell types. The first images confirm co-localization of Pd and S by this method, where the areas of high Pd and S correspond to single cells (Supplementary Figure S7B); numerical analysis of the data to gain whole-population correlations of co-localization of Pd, Ru as well as S, P, and N is in progress.

### Analysis of Cell Surfaces by X-Ray Photoelectron Spectroscopy (XPS)

**FIGURE 8:**
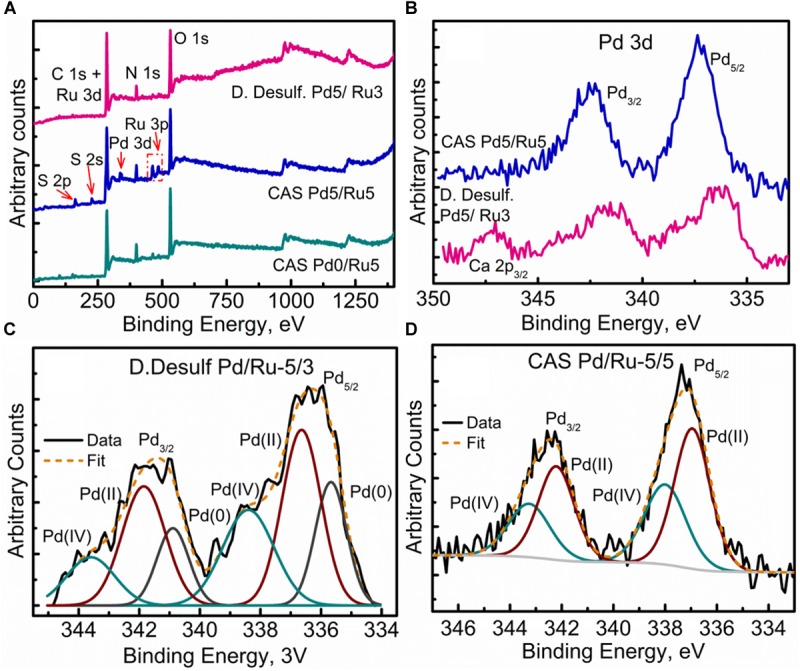
XPPS analysis showing **(A)** Wide energy survey spectra for the metalized bacterial samples, **(B)** High-resolution Pd 3d comparison for the two bimetallic samples, **(C)** Pd 3d fitted components for the *D. desulfuricans* Pd5/Ru3 sample, and **(D)** Pd 3d fitted components for the CAS Pd5/Ru5 sample.

XPS analysis was carried out on the metallized *D. desulfuricans* (Pd5%/Ru3%) and the metallized CAS samples (Pd0%/Ru5% and Pd5%/Ru5%). Wide energy survey spectra recorded for the three types of samples (Figure 8A) identified the presence of C 1s +Ru 3d along with N 1s, O 1s and S 2p. In addition, a small signature of Pd 3d and Ru 3p was also noted for the *D. desulfuricans* material. In the CAS samples, apart from the above, S 2s and S 2p were identified. Bimetallic CAS consisted of a higher sulfur content (3.55 at%) than CAS (Ru5%), which revealed 1.18 at% sulfur content (see Table 3) from the elemental composition obtained from XPS, despite the similar wash procedures applied in each case. The production of H_2_S by washed, resting cells was not quantified but may have arisen from protein turnover/degradation as sulfate was not supplied to the resting cell suspensions (see above Discussion). Bimetallic CAS also confirmed the presence of Pd, identified as Pd 3d as well as a higher Ru (Ru 3p) content. Further detailed analysis of the elements and their chemical interactions was carried out using high-resolution spectra collected for all these elements. A comparison of the high resolution Pd 3d spectra for the two bimetallic systems revealed (Figure 8B) broader doublet peaks for D. *desulfuricans* centered at 336 and 341 eV. The CAS sample, on the other hand, revealed a sharper doublet peak shifted to higher binding energy (337, 342 eV). Deconvolution of the two spectra into respective components can be seen in Figure 8C,D, respectively. *D. desulfuricans* samples consisted of Pd in its metallic [Pd (0)] as well as oxidized forms [Pd(II) and Pd(IV)], while the bimetallic CAS with its spectra shifted to higher binding energies suggested a complete absence of metallic Pd and consisted of Pd only in its oxidized forms. The presence of palladium sulfides (Pd_x_S_y_) which may have been formed due to exposure of H_2_S during the growth of NPs cannot be ruled out in this case. This would further justify the shift in the Pd spectra and explain the complete absence of Pd in its metallic form (unlike previous reports on bio-Pd), whereby any unoxidized Pd NPs were “claimed” by the sulfur. The signature binding energies for (Pd_x_S_y_) in the high resolution Pd spectra are similar to those of oxidized Pd (Xu et al., 2013) and therefore, could not be identified as separate components here.

**FIGURE 9:**
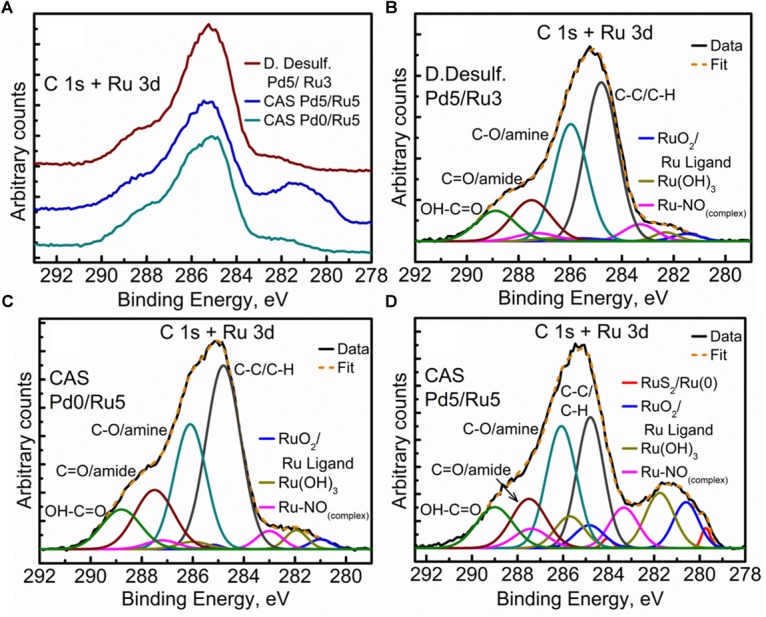
High Resolution C 1s + Ru 3d spectra showing **(A)** comparison of the three metallized bacterial samples, **(B)** fitted components for the *D. desulfuricans* Pd5/Ru3 sample, **(C)** fitted components for the CAS Pd0/Ru5 sample, and **(D)** fitted components for the CAS Pd5/Ru5 sample.

The high resolution C 1s + Ru 3d spectra for the three samples are compared in Figure 9A (comparison of Ru 3p spectra can be seen in Supplementary Figure S8). Here again, bimetallic CAS shows a significantly larger “bump” with a peak centered near 281 eV, attributable to the higher amount of Ru content as compared to other two samples. It must be noted that the Ru 3d_5/2_ region extends to 279 eV in case of bimetallic CAS unlike *D. desulfuricans* and Ru-only CAS where the signal was observed only up to 280 eV. Component peaks for the three samples can be seen in the deconvoluted spectra in Figure 9B–D. Components identified in the three spectra were similar to those reported in similar bacterial systems reported earlier (Priestley et al., 2015; Gomez-Bolivar et al., in review). In the case of bimetallic CAS, the extended Ru 3d_5/2_ region (in which the Ru components are more easily identifiable compared to Ru 3d_3/2_ which is overshadowed by C 1s components), suggested the presence of an additional Ru component near 279.7 eV. This component is close to the binding energy (BE) of metallic Ru and RuS_2_. The component is more likely RuS_2_ in this case given: (i), the presence of H_2_S produced by the bacteria during the NP synthesis; (ii), metallic Ru is highly unlikely to be present in the oxidizing environment near the surface layers of the bacteria given its oxophilic nature, as reported previously (Gomez-Bolivar et al., in review; this volume), and (iii), this is agreement with the presence of sulfide in the Pd (and S2p) spectra, emphasizing the sulfidation of the available metal NPs taking place in this system.

**Table 4:** Binding energies for the components identified in sulfur spectra along with peak attributions.

Component	Binding Energy, eV		Attributions	References
	CAS Ru 5%	CAS Pd5%/Ru5%		
S1	162.5, 163.7	162.5, 163.8	RuS_2_, Pd_x_S_y_	De Los Reyes et al., 1990; Love et al., 2003
S2	163.9, 165.1	163.9, 165.1	-S-S-, S = C = S, -S-CH_3_	Lindberg et al., 1970; Love et al., 2003
S3	166.3, 167.5	166.4, 167.6	SO_2_-Na, aromatic -C-S-O-,	Lindberg et al., 1970
S4	168.2, 169.4	168.2, 169.4	Sulfided Pd/C, SOCl_2_	Lindberg et al., 1970; Xu et al., 2013

**FIGURE 10:**
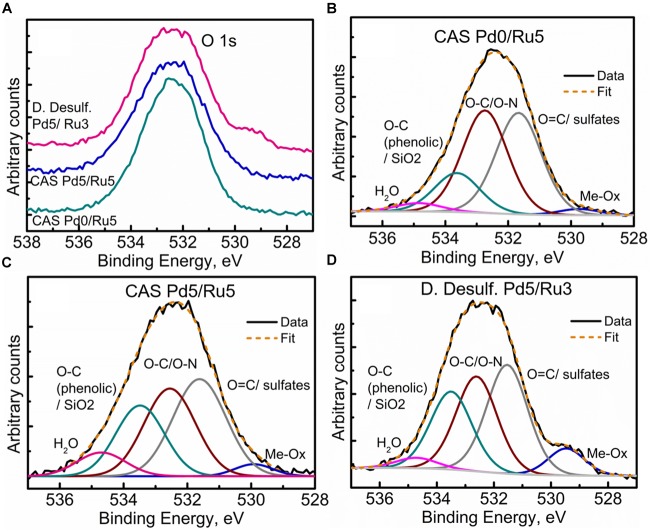
High Resolution O 1s spectra showing **(A)** comparison of the three metallized bacterial samples, **(B)** fitted components for the CAS Pd0/Ru5 sample, **(C)** fitted components for the CAS Pd5/Ru5 sample fitted, and **(D)** components for the *D. desulfuricans* Pd5/Ru3 sample.

Further clarity in the sulfidation/oxidation of the metal NP in these bacterial systems was attained with the help of high-resolution O 1s spectra for the three systems (Figure 10A–D). A simple comparison of the high resolution O 1s spectra (Figure 10A) revealed a clear shoulder below 530 eV in the bimetallic *D. desulfuricans*, suggesting a higher metal oxide content. The deconvolution of the three spectra revealed typical peaks attributed to metal oxide (Me-Ox), O = C/sulfate, O-C/O-N, phenolic O-C/SiO_2_, and adsorbed H_2_O. As seen in Figure 10D, the Me-Ox component for bimetallic *D. desulfuricans* in O 1s spectra has a much higher contribution as compared to that in the other two samples. Looking back at the elemental compositions, the atomic % of oxygen and ruthenium was similar in the material of *D. desulfuricans* and Ru-CAS (Table 3) yet *D. desulfuricans* has higher Me-Ox component. Bimetallic CAS consisted of a slightly higher Ru content than the other two samples along with a higher S content. Hence, it can be concluded that the CAS samples must consist of Me-Ox as well as metal sulfides (for Pd and Ru) and that metallic Ru is unlikely to be present in the surface layers of the bacterial systems observed in the XPS.

**FIGURE 11:**
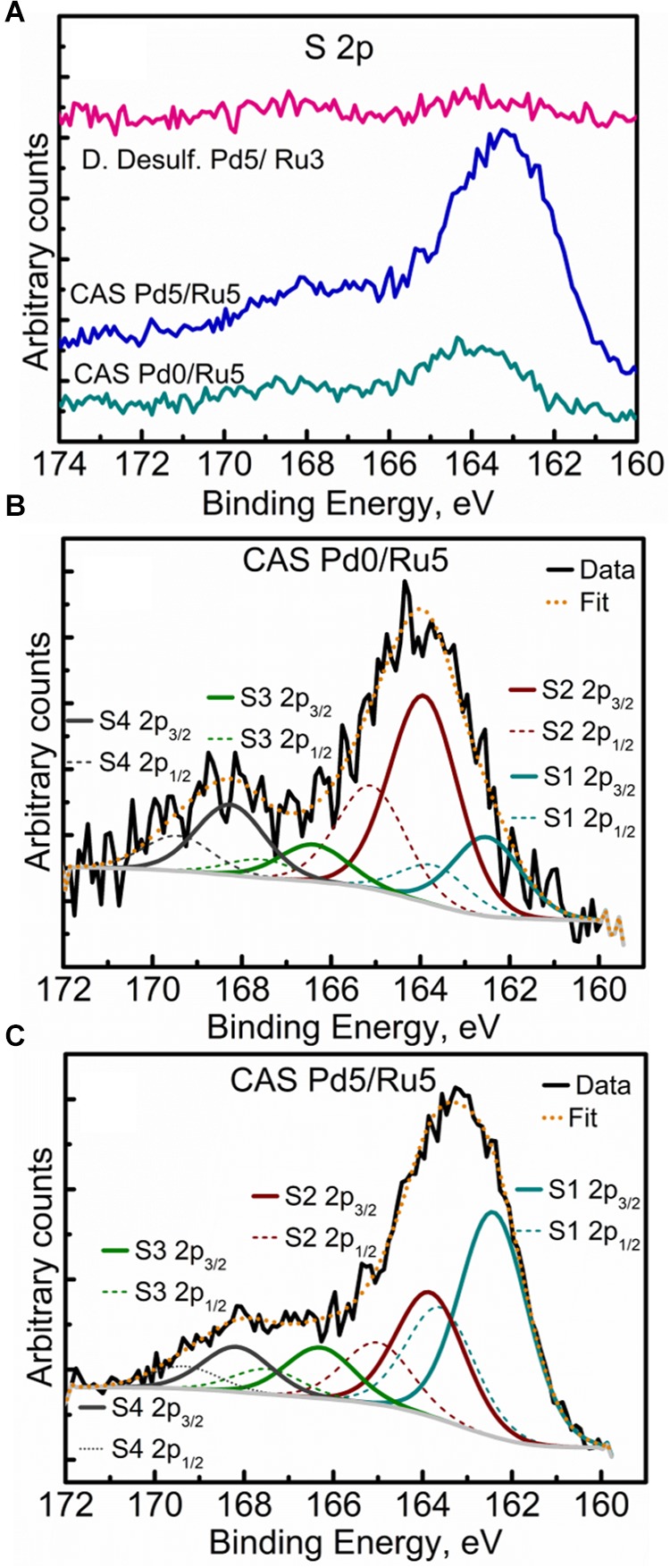
High Resolution S 2p spectra showing **(A)** comparison of the three metallized bacterial samples, **(B)** fitted components for the CAS Pd0/Ru5 sample, and **(C)** fitted components for the CAS Pd5/Ru5 sample.

High-resolution S 2p spectra are seen in Figure 11A–C (see Supplementary Figure S9 for comparison of 2p spectra). Bimetallic CAS sample revealed a peak centered near 163 eV while the Ru-CAS sample showed a peak centered near 164 eV. Deconvolution of the two revealed four sets of components (2p doublets, 2p_3/2_, and 2p_1/2_), identified as S1, S2, S3, and S4. These component peaks were identified with 2 or more attributions, as listed in Table 4. The primary reason for these multiple attributions is the complex nature of these metallized CAS and *D. desulfuricans* samples as well as the similar omnipresent sulfur present in the form of H_2_S, which was produced by the bacteria themselves. Various S-C bonds, SOx-Me bonds, and S-C-Me (Me = Ca, Fe, Na, Cl, F, i.e., trace metals naturally present in bacteria) complexes are possibly formed due to the interactions between polymeric/aliphatic/aromatic carbon structures within the bacterial structures and the available sulfur. The binding energies (BEs) for such bonds and complexes are very close to each other and hence single attributions to thee sulfur components cannot be identified in these systems. Similarly, the BEs for RuS_2_ and Pd_x_S_y_ are also close to reach other and components cannot be identified individually. Interestingly, bimetallic CAS with higher S at%, appears to have a higher metal sulfide component S1 which is in agreement with the Ru 3d and Pd 3d spectra.

## Conclusion

This study shows clear potential for the harnessing of biomass waste side-streams into additional energy materials (via bio-NP catalysts) to help offset the “parasitic” energy demand of biomass comminution and hydrothermal processing. A common solvent allowed a single stage processing, extraction and catalytic upgrading of 5- HMF to make DMF, a “drop in” fuel. Commercial catalyst, although effective in upgrading of commercial 5-HMF, had low activity against 5-HMF derived from thermochemical processing of starch and cellulose. Pd/Ru bimetallic nanoparticles made and supported on bacterial cells were effective in this reaction. Waste sulfidogenic bacteria from another, unrelated, biotechnology process outperformed the “classical” sulfidogen *D. desulfuricans*. This may be attributed to the higher Ru content of the bimetallic of the former but could equally well be assigned to the higher proportion of metal sulfides formed in resting cells of the bacterial consortium without exogenous oxidized sulfur species. The role of palladium sulfide ensembles as enhanced hydrogenation catalysts is just emerging in the literature; as yet a role is not assigned for ruthenium sulfides in hydrogenation such as we suggest. The contributory roles of Pd(0) and oxidized Pd species, palladium sulfides and the various species of Ru (III) (IV) (VI) and RuS_2_, await further elucidation via advanced characterization methods. However, the use of mixed metal NPs opens new opportunities for using metals recovered for wastes, as waste streams rarely contain single metals; neo-catalyst bio-genesis from waste is now well established in other published work.

## Author Contributions

Biomaterials were made and characterized by JG-B and IM. RO developed the method for 5-HMF extraction from hyrolyzates and common reaction solvent system and made the energy balance calculations. RO and JG-B did catalytic testing, with analysis of products by RH. SEM and high resolution TEM/elemental mapping were done by JG-B and MM. XPS data acquisition was done by MW with XPS interpretations by MW and SS. Synchrotron measurements were done by MM, IM, and LM with interpretation by MM. DJ and BG maintained the SCW culture and provided the samples of CAS for use in this study. The paper was authored by LEM with contributions from all authors.

## Conflict of Interest Statement

The authors declare that the research was conducted in the absence of any commercial or financial relationships that could be construed as a potential conflict of interest.

## Footnotes

1 http://www.diamond.ac.uk/Beamlines/Spectroscopy/IO8.html

2 https://www.diamond.ac.uk/Instruments/Imaging-and-Microscopy/I08
