# Supplementary material for: Upconversion of Cellulosic Waste Into a Potential “Drop in Fuel” via Novel Catalyst Generated Using Desulfovibrio desulfuricans and a Consortium of Acidophilic Sulfidogens
Source: Front Microbiol. 2019 May 10;10:970. doi: 10.3389/fmicb.2019.00970 (PMC6523789; doi:10.3389/fmicb.2019.00970)

Figure S1A Example GC chromatograms of products from hydrothermal hydrolysis of starch @ 220 ^{o}C (top) and cellulose @ 260 ^{o}C (bottom)

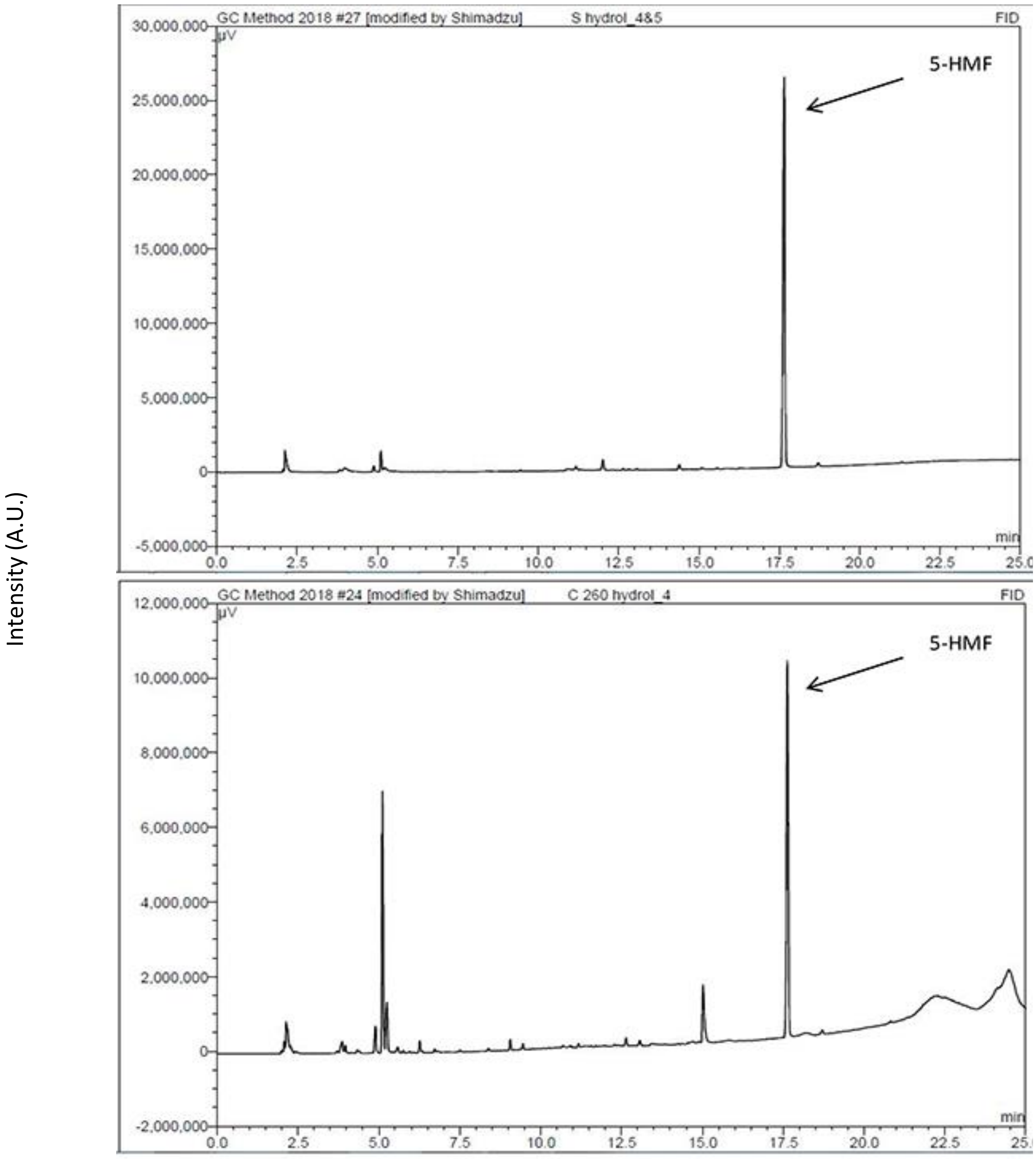

Figure S1.B Example chromatograms of products from catalytic upgrading of 5-HMF

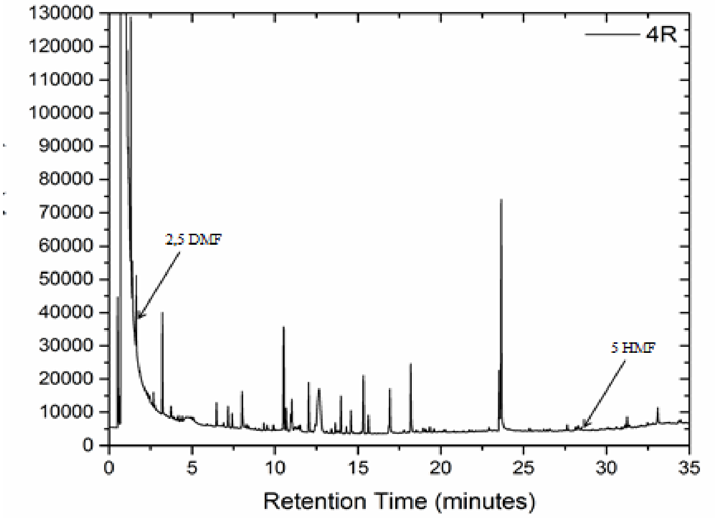

Products from 5-HMF upgrading using 5wt% Ru-C commercial catalyst and commercially obtained 5-HMF (46 mM DMF, no 5-HMF).

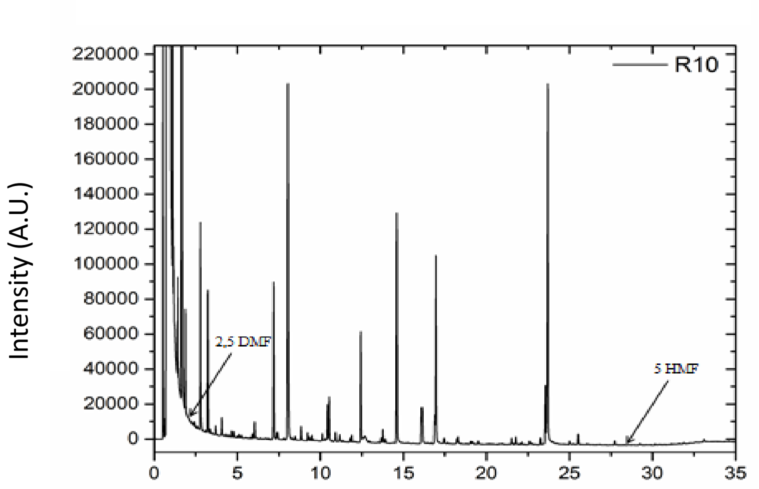

Products from 5-HMF upgrading using 5wt% Ru-C catalyst and 5-HMF obtained via starch hydrolysis (No DMF, no 5-HMF).

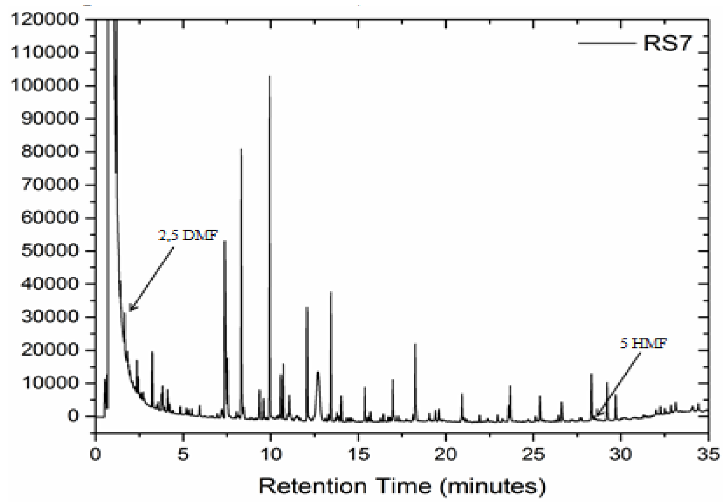

Products from 5-HMF upgrading using Pd/Ru catalyst on CAS and 5-HMF obtained via starch hydrolysis (20 mM DMF, 0.2 mM 5-HMF)

Table S1A. Comparative hydrogenation reactions converting commercial 5-HMF to DMF under two catalyst loadings and different reaction conditions in MTHF and THF as solvents

	MTHF			THF		
Catalyst	DMF (mM)	5-HMF (mM)	Reaction conditions	DMF (mM)	5-HMF (mM)	Reaction conditions
5% Ru-C	45.7	0	100 mg cat; 260 °C; 50 bar	16.7	0	50 mg cat; 260 °C; 50 bar
5% Ru-C				12.4	2.9	100 mg cat; 200 °C; 20 bar
Bio-5%Pd/5%Ru	9.0	6.4	100 mg cat; 200 °C; 20 bar	0.25	52	100 mg cat; 200 °C; 20 bar
Bio-5%Pd/5%Ru	15.1	1.5	50 mg cat; 260 °C; 50 bar	5.1	14	50 mg cat; 260 °C; 50 bar
No catalyst	0.6	74.2	200 °C; 20 bar	0	74	200 °C; 20 bar

The reaction volume was 25 mL in al reactions and the reaction time was 2h. In all cases the conversions to DMF are higher with MTHF compared to THF suggesting that MTHF is a better reaction solvent under these conditions.

Table S1B. Yields of main product 2,5 DMF for the different catalytic reactions and 5-HMF sources (set 1, 2 and 3 as described). Values are average of 2 experiments ± S.E.

Catalyst	**5-HMF commercial (set1)**			**5-HMF starch (set2)**			**5-HMF cellulose (set3)**		
	5-HMF	2,5 DMF		5-HMF	2,5 DMF		5-HMF	2,5 DMF	
	Convers	Yield %	Select	Convers	Yield %	Select	Convers	Yield %	Select
Pd/Ru DD*	100	35.88 ± 2.3	35.88 ± 2.3	97.8	13.23 ± 1.1	13.53 ± 1.1	73.38 ± 6.8	16.19 ± 1.4	22.06 ± 1.9
Pd/Ru CAS**	99.88	63.13 ± 4.1	63.2 ± 4.1	**98.54**	**23.64 ± 0.8**	**23.99 ± 0.9**	**68.71 ± 6.5**	**16.67 ± 1.7**	**24.26 ± 2.5**
5%Ru CAS	100	33.75 ± 0.9	33.75 ± 0.9	99.84	29.3 ± 2.5	29.33 ± 2.5		Not done	
5%Ru $_{Comm.}$	100	**57.06 ± 3.8**	**57.06 ± 3.8**	100	0 ± 0	0 ± 0	100	3 ± 0.29	3 ± 0.3
5%Pd $_{Comm.}$	100	6.44 ±0.75	6.44 ± 0.75	100	2.16 ±0.42	2.16 ± 0.4		Not done	

* DD: *Desulfovibrio desulfuricans;*
** CAS: consortium of acidophilic sulfidogens Pd/Ru on bacteria was 5wt%Pd/5wt%Ru.
5%Ru$_{Comm}$: commercial 5wt%Ru on carbon catalyst.
5%Pd$_{Comm}$: commercial 5%wtPd on carbon catalyst
Convers: conversion (%); Select: Selectivity to 2,5 DMF (%)
Note lack of activity of commercial catalysts to obtain 2,5-DMF from 5-HMF in starch/cellulose hydrolysates, whereas bio-derived catalyst achieved selective conversion to 2,5-DMF.

Figure S2. Pd deposition by *D. desulfuricans* at 20 wt% loading onto cells.

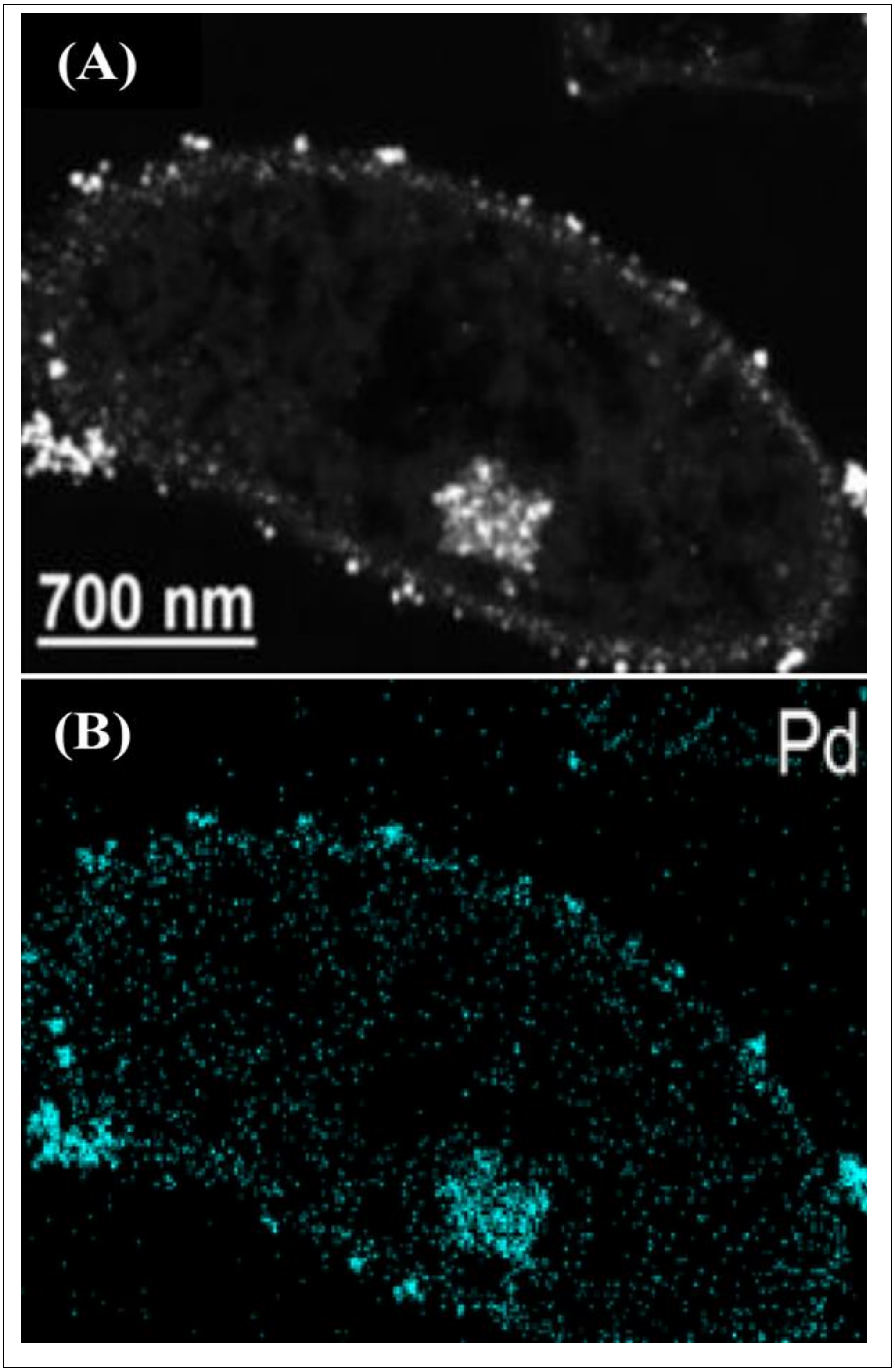

Figure S3. HAADF-STEM micrographs of Pd(0) on cells of *E. coli* (A) and *D. desulfuricans* (B) at 5wt% loading onto biomass, with elemental mapping for Pd, P and S.

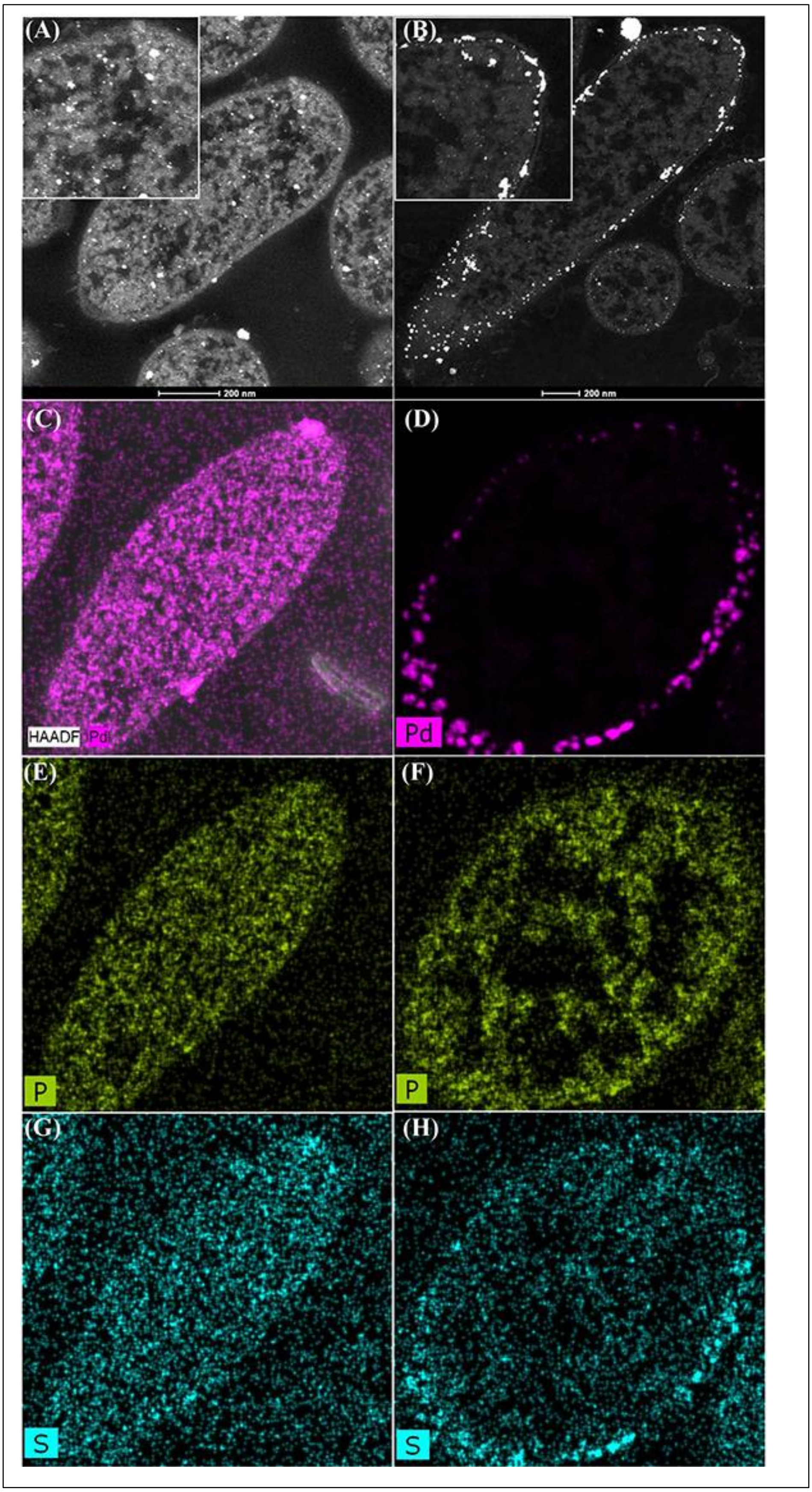

Figure S4. STEM/HAADF images of *D. desulfuricans* loaded with 5%wtPd/3wt%Ru

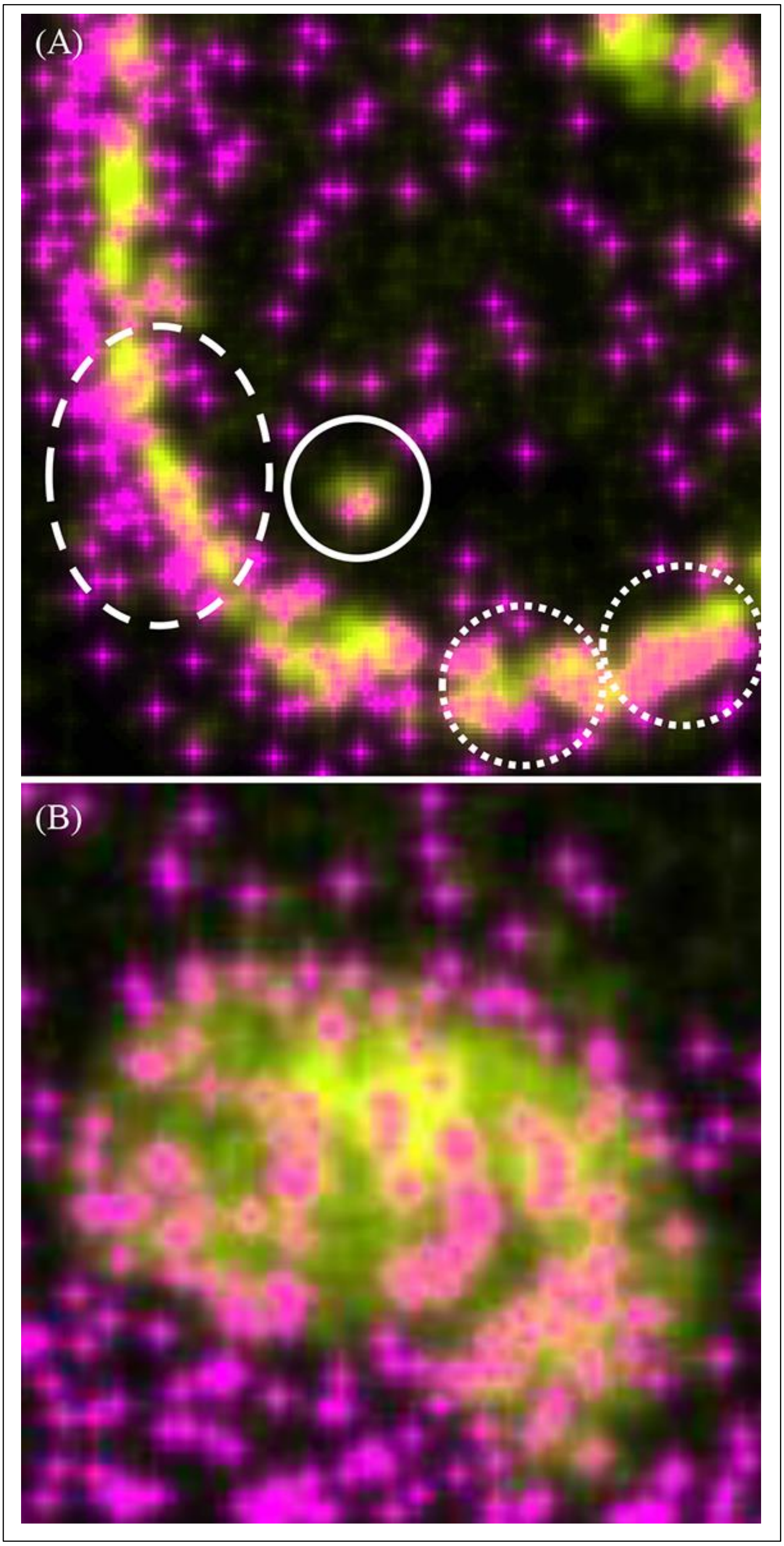

Figure S5. Consortium of acidophilic sulfidogenic bacteria (CAS) viewed by SEM (A) and images of cells loaded to 5wt%Pd/5wt%Ru (B,C).

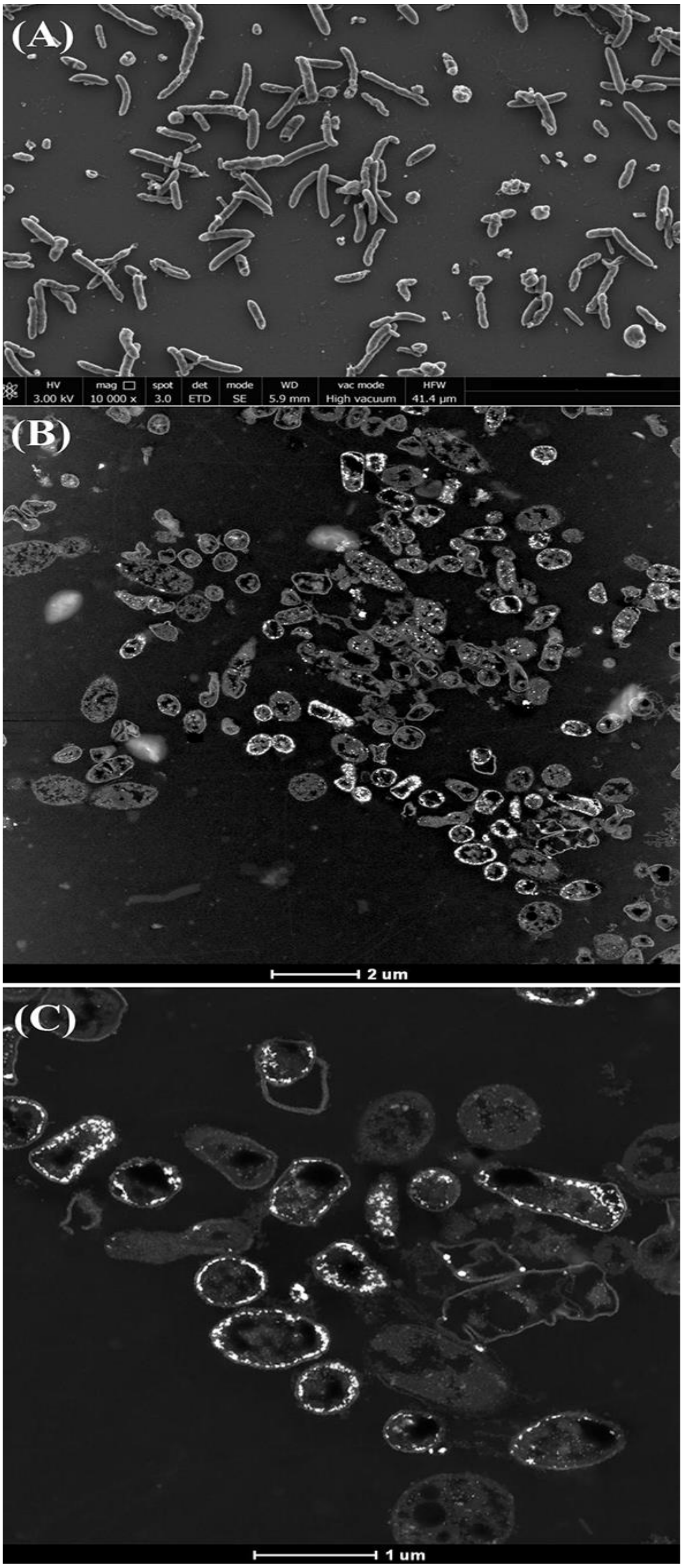

Figure S6. Four types of metal-accumulating bacteria within the CAS, loaded to 5wt%Pd/5wt%Ru

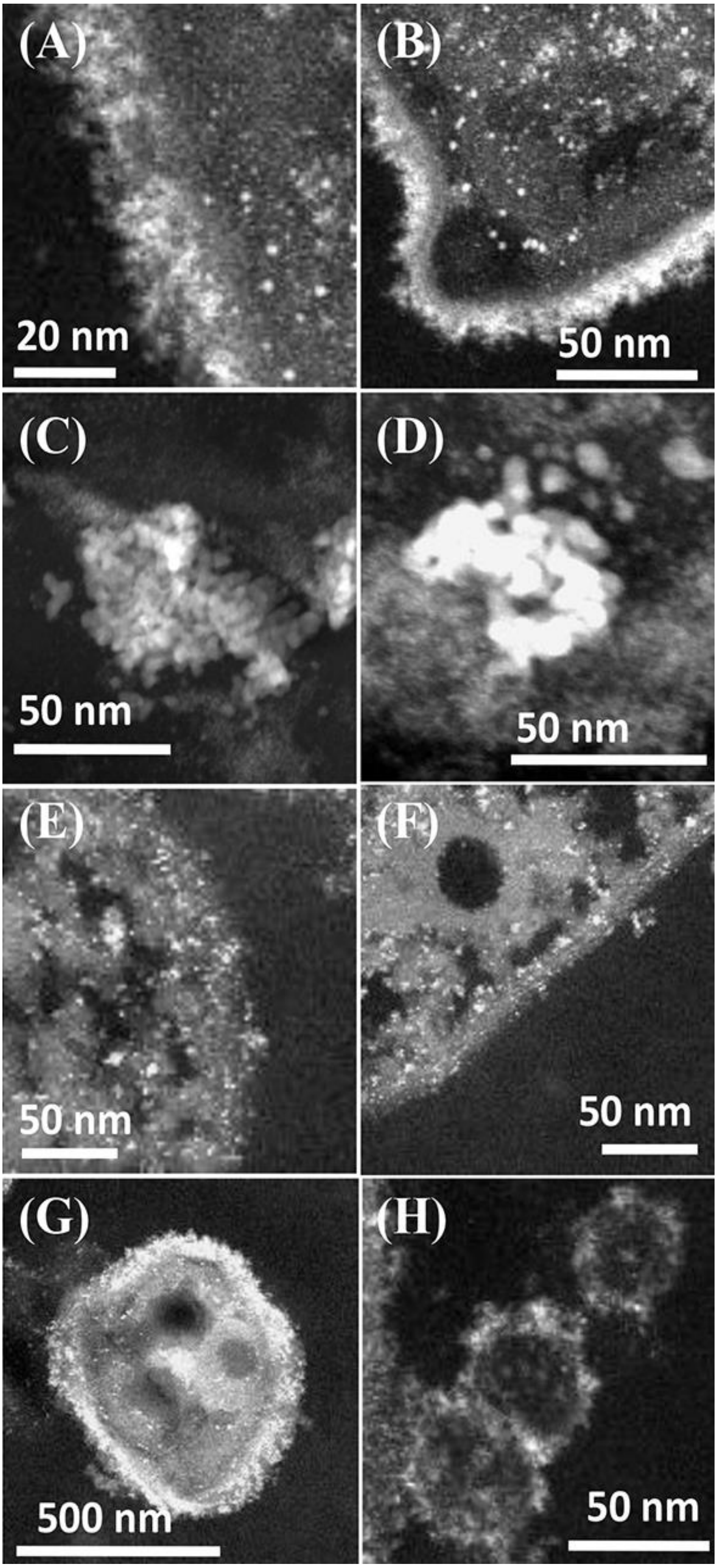

A,B: surface layers of CAS Type I cells

C,D: surface layers of CAS Type II cells

E,F: surface layers of CAS Type III cells

G: CAS Type IV cells (probable spore)

H: Extruded outer membrane vesicles from a type I cell

Figure S7A. Expanded regions of cell surfaces of CAS bacteria loaded with 5wt%Pd/5wt%Ru

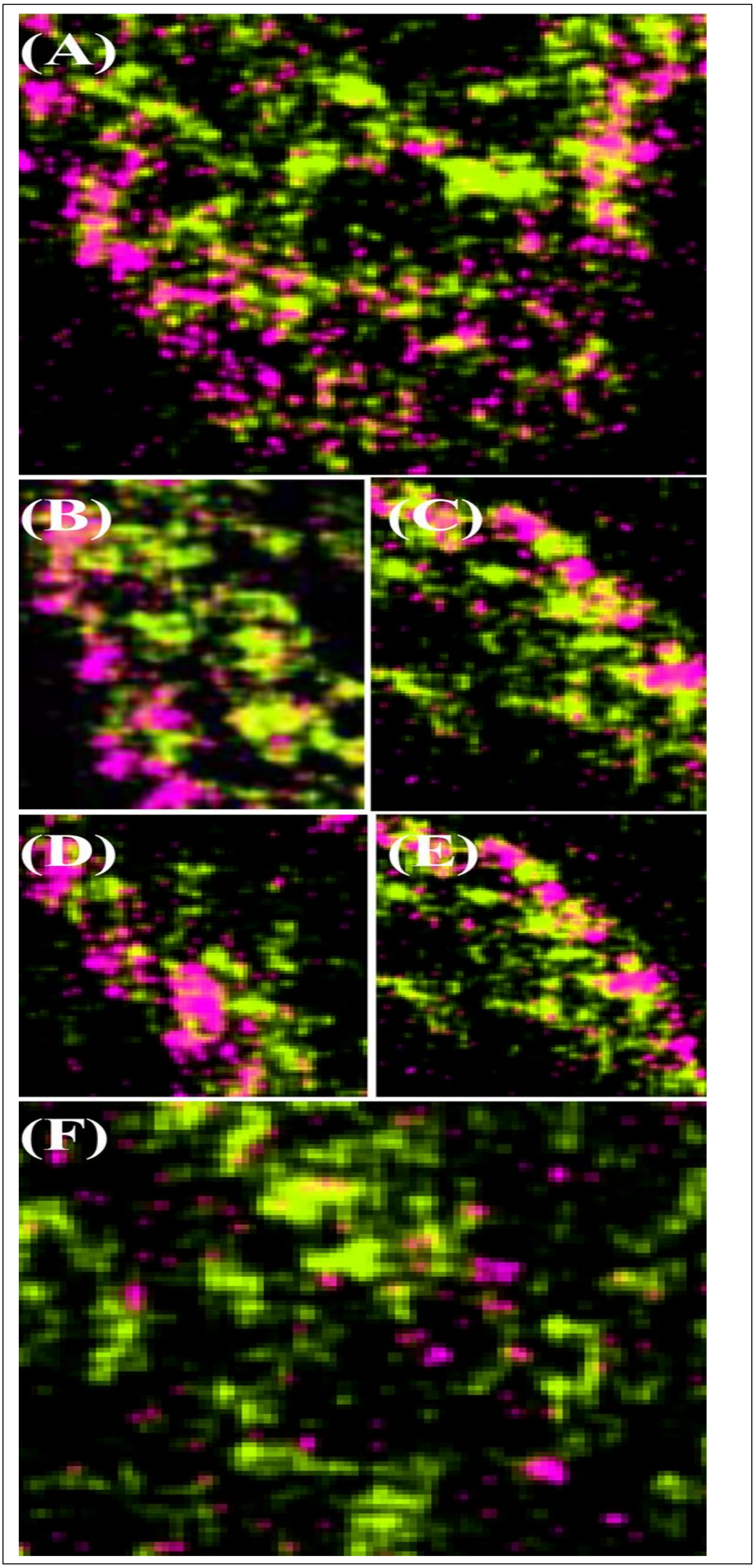

Figure S7B. Preliminary data showing use of X-ray microscopy via synchtrotron radiation to promote element-specific X-ray emission

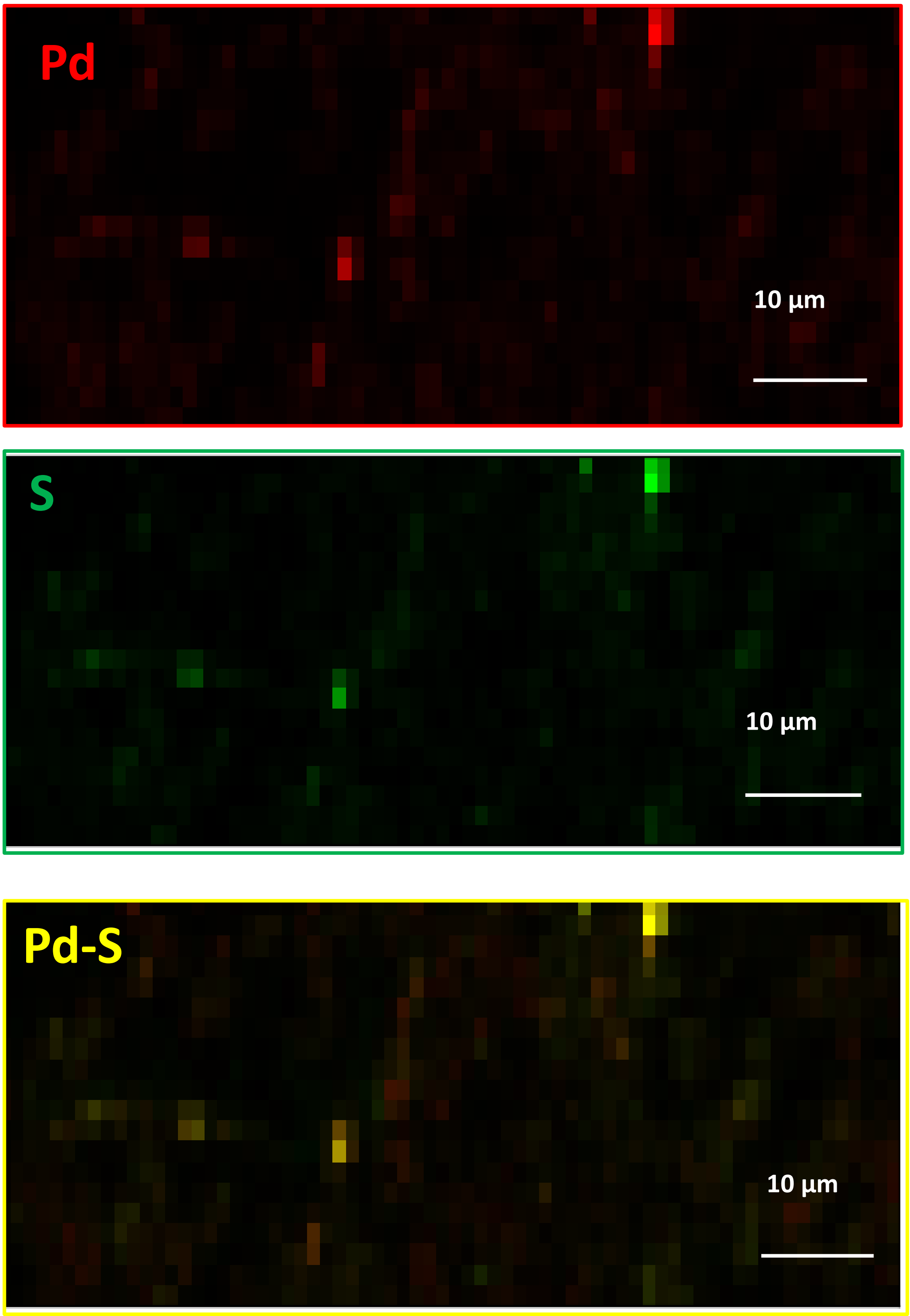

Figure S8. Ru3p XPS spectra for 3 biometallic samples

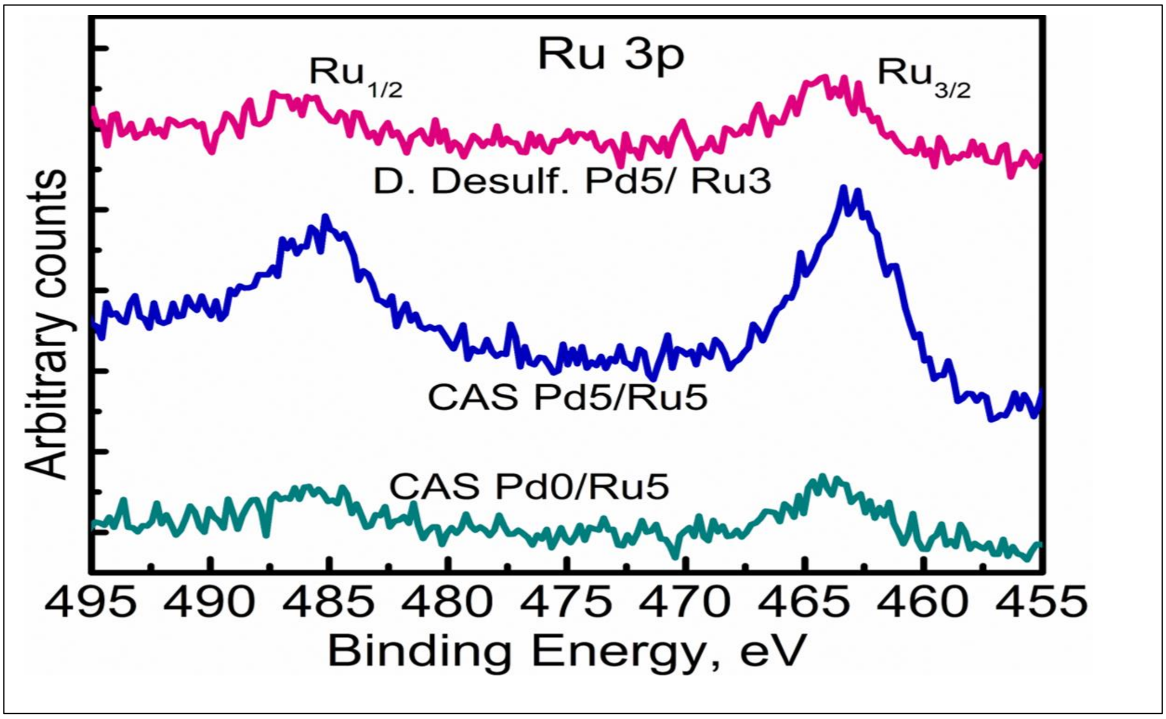

Figure S9. P2p XPS spectra for 3 biometallic samples

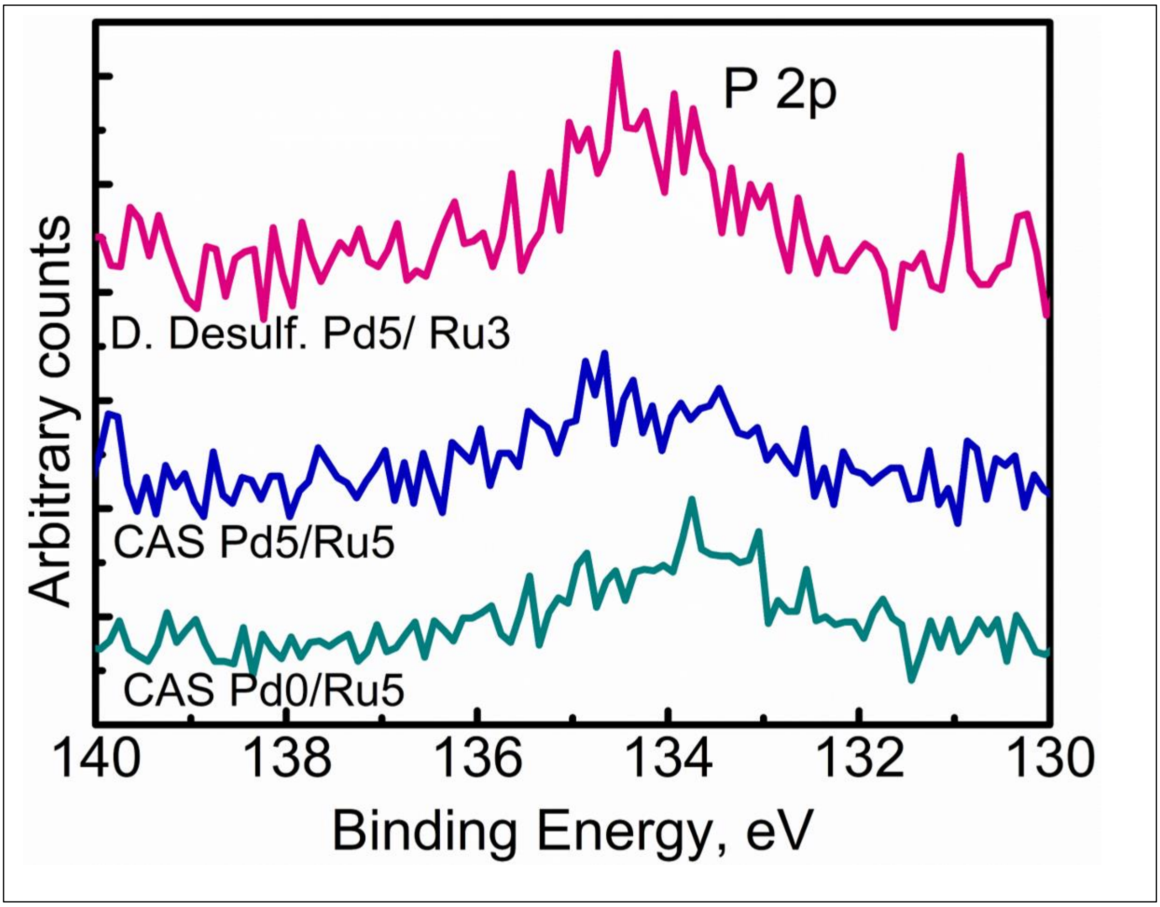

Supplement: Supplementary file 1 [file Data_Sheet_1.PDF]
